# Thermodynamic Consideration of the Solid Saponin Extract Drop–Air System

**DOI:** 10.3390/molecules28134943

**Published:** 2023-06-23

**Authors:** Adam Grzywaczyk, Wojciech Smułek, Ewa Kaczorek, Anna Zdziennicka, Bronisław Jańczuk

**Affiliations:** 1 Institute of Chemical Technology and Engineering, Poznań University of Technology, Berdychowo 4, 60-965 Poznań, Poland; adam.grzywaczyk@doctorate.put.poznan.pl (A.G.); wojciech.smulek@put.poznan.pl (W.S.); ewa.kaczorek@put.poznan.pl (E.K.); 2 Department of Interfacial Phenomena, Institute of Chemical Sciences, Faculty of Chemistry, Maria Curie-Skłodowska University in Lublin, Maria Curie-Skłodowska Sq. 3, 20-031 Lublin, Poland; anna.zdziennicka@mail.umcs.pl

**Keywords:** contact angle, adsorption, saponins, adhesion tension, adhesion work, Gibbs free energy of adsorption

## Abstract

The aim of this research was to elucidate the surface active properties of *Saponaria officinalis* (soapwort) extract containing the plant surfactants saponins. To this end, the advancing contact angle (θ) of water, formamide and diiodomethane on the glass, as well as θ of the aqueous solution of *S. officinalis* extract fractions on PTFE, PMMA and glass, were studied. Based on the obtained results, the wetting behaviour of saponins was considered with regard to the surface tension components and parameters of the solutions and solids. The investigations also involved the description of the θ isotherms, the dependences between the cosine of contact angle and/or the adhesion of the solution to the solid surfaces and solution surface tension, as well as the critical surface tension of PTFE, PMMA and glass wetting. These dependences were studied based on the saponin adsorption at the different interfaces, which was deduced from the dependence between the adhesion and surface tension of the solution, as well as using the Gibbs and Frumkin isotherm equations. This proved that the saponins are poor wetting agents and that the contact angle isotherm can be described by the exponential function of the second order as well as the Szyszkowski equation, but only for PTFE.

## 1. Introduction

The popularity and application of natural surfactants has been growing in recent years due to increasing environmental pollution with synthetic surfactants and detergents. Saponins, defined as steroidal or triterpene glycosides, are unique representatives of biosurfactants. They originate from plants; hence, it is possible to isolate them in relatively high quantities, especially in comparison to bacterial biosurfactants. Saponins have found use in various fields, for example, in the food industry, pharmacy, medicine, cosmetics and agriculture, and the application areas are growing steadily [1,2,3,4,5,6]. In recent years, there has been great interest in the use of naturally occurring compounds used in oral hygiene products, and for their inclusion into traditional therapeutic and orthodontic procedures. Saponins are characterized by their anti-inflammatory, antibacterial, antiviral, protozoal and antifungal properties, and can be also used as additional agents in the dental implantology [7,8]. Studies have also proved that extracts containing saponins have a suppressive impact on bacteria such as *S. mutants* [9].

The interest in saponins, and surfactants in general, is closely related with their ability to form micelles and their ability to adsorb at different interfaces, which is associated with the wetting property of their solutions [10,11,12].

Despite numerous studies, the issues associated with wetting properties have not been completely solved, not only in case of aqueous surfactant solutions, but even for pure liquids [13,14,15,16,17,18,19,20,21,22,23,24,25,26,27,28,29]. Following the rules of thermodynamics, the wetting properties of a given liquid and/or solution depend on the difference between its adhesion to the solid surface and the cohesion. The complete spreading of the liquid and/or the solution over the solid surface occurs if the adhesion is equal to or higher than the cohesion. However, to date, there have been different opinions in the literature regarding the value of the liquid or solution surface tension for which the adhesion to the solid surface is equal to the cohesion, as well as about the relationship between this value and that of the solid surface tension [17,18,22,25]. Such a value of the surface tension of the liquid or the solution was called the critical surface tension of the solid wetting ($\gamma_{C})$ by Zisman [13]. Based on the investigations of the given solid wettability based on the contact angles of different series of liquids, and the Young equation, the conclusion was drawn that for a given solid, the $\gamma_{C}$ value depends not only on the type of liquids used for its determination, but also on their total surface tension and the type of intermolecular interactions contributing to this tension [16,17,18,22,23,24,25,26,27,28].

Van Oss et al. [18,20] modified the Fowkes concept [17] by dividing the solid and liquid surface tension into the Lifshitz–van der Waals (LW) component and the acid–base (AB) component, which is a function of the electron-acceptor and electron-donor parameters. According to the contribution of given intermolecular interactions to the surface tension of liquids and solids, they can be treated as apolar, monopolar or bipolar. Hence, the wetting of a solid depends on the values of the LW component and the electron-acceptor and electron-donor parameters of liquid and solid surface tension, as well as the relation between the LW and AB components. This applies when the contact angle is greater than zero, as it can be assumed that the liquid layer adsorbed on the solid surface does not change its surface tension and the liquid molecules do not form the surface layer at the solid–liquid interface characterized by the specific properties. This situation is more probable in the case of single liquids characterized by small molecules than, for example, the aqueous solutions of surfactants. The surface tension of surfactants depends on the orientation of their molecules toward the air phase, and they can be oriented in different ways at different interfaces [30].

In the case of aqueous solutions of multicomponent mixtures of surfactants as well as surfactants with additives, it can be expected that their wetting properties depend on many parameters, and it is difficult to find investigations on the wetting properties of such solutions in the literature. Hence, the aim of our study was to determine the wetting properties of the aqueous solution of different types of extracts from *Saponaria officinalis*, which include surfactants and other compounds using apolar (polytetrafluoroethylene (PTFE)), monopolar (polymethyl methacrylate (PMMA)) and bipolar (glass) solids. The advancing contact angles of their aqueous solutions on the surface of selected solids were measured in order to determine the wetting properties of these extracts. Based on the obtained results, the wetting process was investigated with regard to the adsorption of the extract components at the water–air (W-A), solid–water (S-W) and solid–air (S-A) interfaces, as well as to adhesion of the solution to solids. The standard Gibbs free energy of adsorption at these interfaces was also taken into account.

## 2. Results and Discussion

### 2.1. Contact Angle

In 1805, Thomas Young established that the contact angle (θ) of a liquid on the solid surface in the air environment depends on the liquid–air ($\gamma_{LV}$), solid–air ($\gamma_{SV}$) and solid–liquid $\left(\gamma_{SL}\right)$ interface tensions. Afterwards, Dupre expressed this statement as the following mathematical form, which is known as the Young equation [12]:

(1) $$\gamma_{SV}-\gamma_{SL}=\gamma_{LV}cos\theta$$

This equation only applies to cases when the vapour of liquid, which can form the layer around its drop settled on the solid surface, does not change the solid surface tension (SST). However, for a given solution, such a layer can be formed not only by the vapour of the component, but also by penetration from the solution drop to the solid surface.

It has been suggested by many authors that if the surface tension (ST) of the given liquid is higher than that of a solid on which the liquid drop was settled, there is no influence of the liquid vapour adsorbed on the solid surface behind the drop on the SST [17,18,25,26,28]. It can be stated that such a case results from Equation (1), as the given solid θ changes only as a function of $\gamma_{LV}$ and $\gamma_{SL}$. On the other hand, it appeared that, for example, θ of diiodomethane and ethylene glycol at 293 K on PTFE is equal to 74.7° and 89.5°, respectively [23,24]. Nevertheless, $\gamma_{LV}$ of diiodomethane is equal to 50.8 mN/m, while that of ethylene glycol is equal to 48.0 mN/m [24]. As follows, a higher surface tension of the liquid (LST) does not guarantee that its θ on PTFE will be higher compared to that of a liquid characterized by lower ST. This fact suggests that θ depends not only on $\gamma_{LV}$ and $\gamma_{SV}$ but also on the contribution of different types of intermolecular interactions to these tensions. Therefore, it seems that the approach of van Oss et al. [18,19,20] to SST and LST as well as to the solid–liquid (S-L) interface tension is useful in terms of the wetting properties of liquids and/or solutions. Thus, taking into account the van Oss et al. approach [18,19,20] and Equation (1), as well as the fact that the layer formed behind the liquid or the solution drop settled on the solid surface by the compounds characterized by $\mathrm{ST}>\gamma_{SV}$ does not change SST, the θ values on the PTFE, PMMA and glass (G) surface can be expressed by the following equations:

(2) $$cos\theta =-1+\frac{2\sqrt{\gamma_{\mathrm{PTFE}}^{\mathrm{LW}}\gamma_{LV}^{\mathrm{LW}}}}{\gamma_{LV}},$$

and

(3) $$\;cos\theta =-1+\frac{2\sqrt{\gamma_{\mathrm{PMMA}}^{\mathrm{LW}}\gamma_{LV}^{\mathrm{LW}}}+2\sqrt{\gamma_{\mathrm{PMMA}}^{-}\;\gamma_{LV}^{+}}}{\gamma_{LV}},$$

and

(4) $$\;cos\theta =-1+\frac{2\sqrt{\gamma_{G}^{LW}\gamma_{LV}^{LW}}+2\sqrt{\gamma_{G}^{-}\gamma_{LV}^{+}}+2\sqrt{\gamma_{G}^{+}\gamma_{LV}^{+}}}{\gamma_{LV}},$$

where LW refers to the Lifshitz–van der Waals interactions; “+” and “−“ refer to the electron-acceptor and electron- donor interactions.

Based on Equation (2), it can be established that the θ changes of the solution on the PTFE surface, as a function of concentration, depend on the surface tension of the solution (LST) and its LW component. Thus, θ=0 if $\gamma_{LV}^{\mathrm{LW}}$ *=* $\gamma_{LV}$ *=* $\gamma_{\mathrm{PTFE}}^{\mathrm{LW}}$*,* which corresponds to the fact that the complete spreading over the PTFE occurs if LST results only from the LW intermolecular interactions and is equal to the PTFE ST, which also results from these interactions.

In turn, the θ changes of the solution as a function of its concentration depend on LST and the electron- acceptor parameter, as well as the LW component of the solution surface tension. The complete spreading of the solution can be observed if $\gamma_{LV}$ *=* $2\sqrt{\gamma_{\mathrm{PMMA}}^{\mathrm{LW}}\gamma_{LV}^{\mathrm{LW}}}+2\sqrt{\gamma_{\mathrm{PMMA}}^{-}\gamma_{LV}^{+}}$.

In the case of glass, the θ changes of the solution as a function of its concentration depend on $\gamma_{LV}$ as well as the parameters and the LW component of this tension. The complete spreading of the solution over the glass should occur if $\gamma_{LV}=2\sqrt{\gamma_{G}^{\mathrm{LW}}\gamma_{LV}^{\mathrm{LW}}}+2\sqrt{\gamma_{G}^{-}\gamma_{LV}^{+}}+2\sqrt{\gamma_{G}^{+}\gamma_{LV}^{+}}$.

When the ST of at least one component of the solution is lower than the PTFE, PMMA or glass surface tension, the left side of Equations (2)–(4) assumes the form $cos\theta +\frac{\pi_{i}}{\gamma_{LV}}$, where $\pi_{i}$ is the surface pressure of i-th component of the solution. As such, the θ of the solution on the PTFE, PMMA or glass also depends on $\pi_{i}$.

It is also possible that during the spreading of the solution drop over the solid surface, the layer composed of one or more substances is formed first, and then the drop does not practically spread over the solid surface but over the solid surface covered by this layer.

### 2.2. Wettability of PTFE

**Figure 1:**
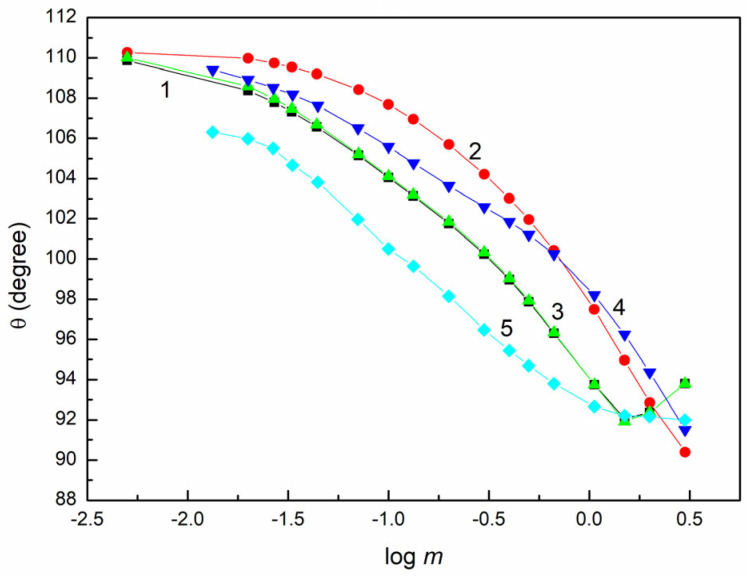
A plot of the contact angle (θ) on the PTFE surface vs. the logarithm of fraction concentration (log m ). Curves 1–5 correspond the fractions E0, E1, E2, E3 and E4, respectively.

The aqueous solution of the individual fractions of saponin is characterized by limited wetting properties in relation to an apolar solid such as PTFE (Figure 1).

The minimal θ value of these solutions is similar to that of ethylene glycol on PTFE [24]. The minimal ST of the aqueous solution of the individual extract fraction is also similar to that of ethylene glycol [24]. The LW component of the ethylene glycol surface tension is equal to 29 mN/m at 293 K. This means that the LW component of the individual fraction of the extract is close to 29 mN/m. This value is similar to that of the surface tension of benzene and other aromatic hydrocarbons. Since the LW component of water surface tension $\left(\gamma_{W}\right)$ is equal to 26.85 mN/m [24,31], the adsorption of the components of the aqueous solution of the extract fraction reduces only $\gamma_{W}^{\mathrm{AB}}$. Thus, the changes of θ in the case of the studied solutions on the PTFE as a function of the solution concentration (C) result from the reduction of $\gamma_{W}^{\mathrm{AB}}$. The aqueous solution of the individual fraction of the extract is a multicomponent system, and its composition is different for the given extract fraction. This is reflected by the shape of the contact angle isotherms (ICA) (Figure 1). It is considerably different from those obtained for Triton X-165 (TX165), sodium dodecyl sulphate (SDS) and hexdecyltrimethyl ammonium bromide (CTAB) (Figure S1) [27]. However, the relationship between θ and $\gamma_{LV}$ of the aqueous solution of both the extract fractions and individual classic surfactants can be described by a single curve (Figure S2). This fact suggests that not only the particular fraction of the extract, but also the classic surfactant, reduces only $\gamma_{W}^{\mathrm{AB}}$ due to the adsorption at the water–air (W-A) interface. In order to confirm this suggestion, θ was calculated based on Equation (2) and assuming that the LW of the aqueous solution of each fraction of the extract is equal to $\gamma_{W}^{\mathrm{LW}}$ (26.85 mN/m) [24,31]. PTFE $\gamma_{SV}$ = 20.24 mN/m [27] was taken into account in this calculation. As a result, a good agreement between the measured and calculated θ values was obtained (Figures S3–S7). This fact confirms our conclusion. In such case, according to the approach of van Oss et al. [18,19,20], the following PTFE–solution interface tension $\left(\gamma_{\mathrm{PTFE},L}\right)$ can be expressed:

(5) $$\gamma_{\mathrm{PTFE},L}=\gamma_{\mathrm{PTFE}}+\gamma_{W}^{\mathrm{LW}}+\gamma_{LV}^{\mathrm{AB}}-2\sqrt{\gamma_{W}^{\mathrm{LW}}\gamma_{\mathrm{PTFE}}^{\mathrm{LW}}},$$

Based on this equation, the changes in $\gamma_{\mathrm{PTFE},L}$ as a function of the extract fraction concentration ($C_{E}$) depend only on those of $\gamma_{W}^{\mathrm{AB}}$. Thus, there is a linear dependence between θ and $\log(\gamma_{LV}+\gamma_{\mathrm{PTFE},L})$ (Figure S8). This relation does not depend on the type of extract fraction.

It is interesting that the ICA of different fractions of extract can be described by the exponential function of the second order (Equation (6)):

(6) $$\theta =y_{0}+A_{1}\exp\left(\frac{-m}{t_{1}}\right)+A_{2}\exp\left(\frac{-m}{t_{2}}\right),$$

where $y_{0},\;\;A_{1}$, $t_{1}$, $A_{2}$. $t_{2}$ are the constants and m is the weight of substrates in g/dm^3^. It can also be described based on the Szyszkowski equation modified for the calculation in the PTFE–solution drop–air system:

(7) $$\gamma_{\mathrm{LV}}\cos\theta -\gamma_{w}\cos\theta_{W}=RTn\Gamma^{max}\ln\left(\frac{m}{a_{1}}+1\right),$$

where $\Gamma^{max}$ is the maximal Gibbs surface excess concentration of the given extract fraction at the PTFE,*L* interface; $a_{1}$ is the constant equal to M ×a (M is the average molar mass; *a* is the constant, which depends on the Gibbs surface free energy of adsorption); *T* is temperature in K; and *R* is the gas constant.

It seems that the constant $y_{0}$ (Table 1) depends on the difference between the LW and AB components of the LST, and it mainly affects the minimum value of θ on the PTFE. However, the other constants most likely influence the shape of the ICA.

Interestingly, in Equation (7) it was assumed that θ changes in the range of $C_{E}$, in which all components of the solution are in the monomeric form. The possibility of the description of θ isotherms as well as $\gamma_{LV}$ of the aqueous solution of the fraction extract can suggest that there is a linear dependence between the adhesion tension (AT, $\gamma_{LV}\cos\theta$) and $\gamma_{LV}$. This suggestion was confirmed by the determined dependences between these magnitudes (Figure S9), which can be expressed by a single linear function for the aqueous solution for all types of extract fractions. In consequence, the slope of this linear function is close to −1, and the other constant in the linear equation that describes this dependence is similar to the adhesion work ($W_{a}$) of the water to PTFE. This fact confirms the reliability of the θ calculations based on $\gamma_{W}^{\mathrm{LW}}$ and the PTFE SF. This also suggests that $\gamma_{LV}$ of any of solution components is not lower than $\gamma_{SV}$ of PTFE.

Based on the linear dependence between $\gamma_{LV}\cos\theta$ and $\gamma_{LV}$, it is possible to determine $\gamma_{C}$ proposed by Zisman [13,15]. The values of $\gamma_{C}$ obtained for PTFE are similar for all saponins extract fractions; however, they are higher than $\gamma_{SV}$ of PTFE (Table 2). This means that the obtained $\gamma_{C}$ values are not as real as the critical surface tension of PTFE wetting. As mentioned above, for such liquid on PTFE, the θ = 0 if $\gamma_{LV\;}$ results only from the LW intermolecular interactions and $\gamma_{LV}=\gamma_{SV}$ of PTFE [27].

If the slope of the linear dependence between AT and LST is equal to −1, then it is possible to determine $W_{a}$ of the solution to the PTFE and to state that this value does not depend on the concentration of the solution. In other words, the linear dependence between AT and LST is applicable only in the range of $C_{E}$, in which the changes in θ take place and $\gamma_{LV}^{\mathrm{LW}}$ is constant. Contrary to the suggestion of Zisman et al. [13,14], there is no linear dependence between cosθ and $\gamma_{LV}$ (Figure S10), as it was stated earlier.

### 2.3. Wettability of PMMA

**Figure 2:**
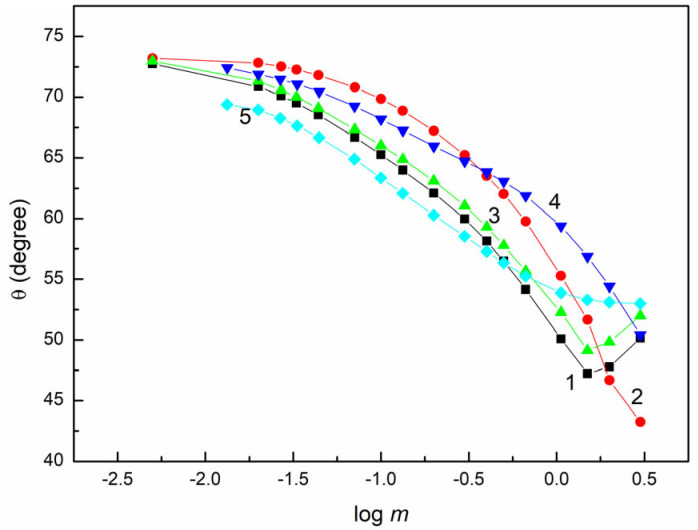
A plot of the contact angle (θ) on the PMMA surface vs. the logarithm of fraction concentration (log m ). Curves 1–5 correspond the fractions E0, E1, E2, E3 and E4, respectively.

The ST of the PMMA (41.28 mN/m) [21,28,29] is more than two times higher than that of PTFE (20.24 mN/m) [21,24,31,32], and PMMA can interact with the adherent medium by the formation of hydrogen bonds because oxygen atoms are present on its surface. As a result, the wetting behaviour of the aqueous solution of various extract fractions is different compared to the PTFE surface (Figure 1 and Figure 2). The minimal θ values are higher than that of ethylene glycol, the ST of which is comparable to the minimal surface tension of the studied solutions (Figure 2) [24]. This suggests that there are compounds present in the particular fraction of the extract characterized by lower ST compared to that of PMMA. They can penetrate from the solution drop to the PMMA behind the drop, thus decreasing its ST. This causes the increase in θ compared to the pure liquids characterized by the same ST as the solution.

It should be highlighted that the ST of PMMA is comparable to that of the head of the sugar surfactants and to other aromatic substances in the solid state [31]. In the studied extract fraction, there are many sugar units and substrates with lower ST compared to PMMA. The θ values of the aqueous solution of the extract fractions on the PMMA are more sensitive to their composition (Figure 2). In contrast to PTFE, the relationship between θ and $\gamma_{LV}$, as well as between θ and $\log\left(\gamma_{\mathrm{PMMA},A}+\gamma_{\mathrm{PMMA},L}+\gamma_{LV}\right)$ (Figure S11), cannot be described by a single function for all studied extract fractions. Furthermore, these functions are not linear.

The investigations regarding the wettability of PTFE by the aqueous solution of different extract fractions showed that $\gamma_{LV}^{\mathrm{LW}}$ and $\gamma_{W}^{\mathrm{LW}}$ do not depend on the concentration and type of extract fraction. This indicates that there should be a linear dependence between $W_{a}$ of the aqueous solution of the extract fraction to the PMMA surface and $\gamma_{LV}$. This dependence can be described by a single linear function for all extract fractions. Such dependence was obtained between $W_{a}$ calculated based on the van Oss et al. equation ($W_{a}=2\sqrt{\gamma_{LV}^{\mathrm{LW}}\gamma_{SV}^{\mathrm{LW}}}+2\sqrt{\gamma_{LV}^{+}\gamma_{SV}^{-}}\;2\sqrt{\gamma_{LV}^{-}\gamma_{SV}^{+}}$) [15,16,17] and $\gamma_{LV}$ (Figure S12). However, in the case of $W_{a}$ calculated based on the Young–Dupre equation (EY-D) ($W_{a}=\gamma_{LV}\left(\cos\theta +1\right)$), there is also a linear dependence between $W_{a}$ and $\gamma_{LV}$; however, it is described by a different linear function for each extract fraction (Figure S12). In all cases, the values of $W_{a}$ determined based on EY-D are lower than those calculated using the van Oss et al. equation [18,19,20]. This fact confirms the conclusions that some substances of the extract fraction with a lower ST than PMMA penetrate from the solution drop settled on the PMMA, which results in a decrease in its ST; and that substances with a different surface tension are present in the given extract fraction.

It seems that the decrease in the PMMA surface tension (π) can be determined based on the difference between $W_{a}$ calculated using the van Oss and EY-D equations ($2\sqrt{\gamma_{LV}^{\mathrm{LW}}\gamma_{\mathrm{PMMA}}^{\mathrm{LW}}}+2\sqrt{\gamma_{\mathrm{PMMA}}^{-}\gamma {\;}_{SV}^{+}}-\gamma_{LV}\left(\cos\theta +1\right)=\pi$). Thus, obtained π values were used for determination of the ST of PMMA covered by the organic substrate layer and the PMMA–solution interface tension $\left(\gamma_{\mathrm{PMMA},L}\right)$.

The θ isotherms of the aqueous solution of the extract fraction on PMMA can also be described by Equation (6) (Figures S13–S17). However, contrary to PTFE, it is difficult to describe such θ isotherms by Equation (7). In contrast to PTFE, the dependence between AT and LST for PMMA cannot be described by a single line (Figure S18). Thus, the $\gamma_{C}$ values depend on the type of extract fractions (Table 2). They are lower not only than the values of the PMMA ST but also than the LW component of this tension [24,27,32].

Similarly to PTFE, the dependence for PMMA between the cosθ and $\gamma_{LV}$ of the solution is not linear (Figure S19). However, the dependence of the $\gamma_{C}$ value for PMMA on the type of extract fraction confirms the statement that $\gamma_{C}$ is not a universal value for a given solid. Additionally, there is no guarantee that the same function describes the θ change in the value for water to zero.

### 2.4. Wettability of Glass

Glass is a bipolar solid due to the presence of oxygen and –OH groups on its surface. The ST depends largely on the type of glass. Therefore, before the θ measurements of the aqueous solution of the extract fractions on the glass, the components and parameters of its surface tension were determined based on θ for water (57.1°), formamide (38.46°) and diiodomethane (45°). Taking into account these values and the components and parameters of the water, formamide and diiodomethane, surface tension [24] the values of the LW component (37.01 mN/m), as well as the $\gamma^{+}$ (1.26 mN/m) and $\gamma^{-}$ (16.15 mN/m) parameters of the glass ST, were obtained and used for considerations of its wettability.

**Figure 3:**
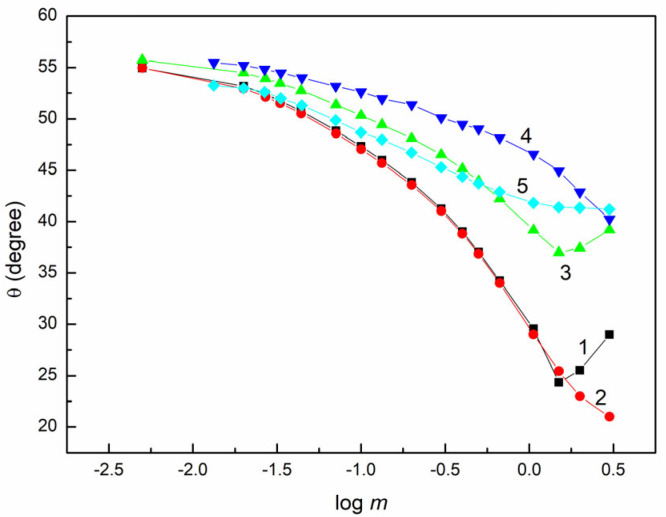
A plot of the contact angle (θ) on the glass surface vs. the logarithm of fraction concentration (log m ). Curves 1–5 correspond the fractions E0, E1, E2, E3 and E4, respectively.

The θ isotherms of the aqueous solutions of E0, E1, E2, E3 and E4 (Figure 3) indicate that these extract fractions have poor glass wetting properties, similar to PTFE and PMMA. The complete spreading of the studied solutions over the glass surface was not observed in any of the studied cases.

While the differences between the ICAs of the aqueous solution of different extract fractions for glass are greater than in the cases of PTFE and PMMA, the isotherms obtained for glass can be described by Equation (6) (Figures S20–S24). However, it is practically impossible to describe these isotherms by Equation (7). In fact, it is difficult to express the constants in Equation (6) with the parameters and components of $\gamma_{LV}$, because many different substrates are present in the solution. It seems that the electrostatic interactions between the molecules of some substrates of the extract fractions and the glass surface affect the θ isotherms. The extract fraction includes organic acids; hence, there are repulsive interactions between their molecules and the glass surface. Therefore, it is probable that there is a positive slope in the dependence between $\gamma_{LV}\cos\theta$ and $\gamma_{LV}$ of the aqueous solutions of E2, E3 and E4 (Figure S25). This indicates weak adsorption of these fractions at the glass–solution (G-L) interface.

For the aqueous solution of E1, there is no linear dependence between the $\gamma_{LV}\cos\theta$ and $\gamma_{LV}$. However, in the case of E0, there is a negative slope in this linear dependence (Figure S25). As a result, the adsorption of the components of the extract fraction at the G-L and glass–air (G-A) interfaces is more complex compared to the systems including PMMA. This conclusion is also confirmed by the dependence between AT and LST (Figure S26). The adsorption of some substrates of the extract fractions at the G-A interface confers the dependence between $W_{a}$ of the solution to glass calculated based on the Young–Dupre [12] and van Oss et al. equations [18,19,20] and $\gamma_{LV}$ (Figure S27). The dependence obtained between $W_{a}$ calculated using the van Oss et al. equation and $\gamma_{LV}$ can be described by a single linear function. However, $W_{a}$ obtained using EY-D can be described by the linear function for each extract fraction separately.

Similarly to PMMA, the difference between $W_{a}$ obtained using two different ways can be treated as π. This pressure reduces the glass surface tension. Based on Figure S27, it can be stated that the reduction in the glass ST depends largely on the type of extract fraction. It should be also noted that in the case of glass, the dependence between contact angle and the logarithm of the sum of the solid and solution surface tensions and solid–liquid interface tension is not linear, and cannot be described by a single function for the studied fractions (Figure S28).

### 2.5. Concentration of the Extract Fractions at the Interfaces

Wettability of the solids by the aqueous solutions of the surfactants depends on the adsorption of surfactants at the solid–air (S-A), solid–solution (S-L) and solution–air (L-A) interfaces and the proper orientation of their molecules in the interface region. The dependence between θ and the adsorption of the surfactants at the interfaces can be expressed by the Lucassen–Reynders equation [33]. It seems that in the particular extract fraction, there is no substance characterized by surface tension lower than PTFE, and no component of the multicomponent mixtures penetrates from the solution drop settled on the PTFE. Thus, it can be assumed that for PTFE, $\Gamma_{SV}=0$ (Γ is the surface excess concentration). For the linear dependence, $\gamma_{LV}\cos\theta =f\left(\gamma_{LV}\right)$ at the slope equal to −1, which takes place in the case of the PTFE–solution drop–air systems (Figure S9), $\Gamma_{SL}=\Gamma_{LV}$ [33]. However, it was not confirmed that the composition of the mixed surface layer is the same at the PTFE,*L* and L-A interfaces.

The concentration of the adsorbed substances at the PTFE,*L* interface can be determined directly for the PTFE–solution drop–air system using the Gibbs isotherm equation, assuming that $\gamma_{SV}$ does not depend on $C_{E}$ and the activity coefficients of all components of the extract fractions are equal to 1 [34].

Since it was difficult to establish the concentration of the ionic substances in the solution, the calculations of $\Gamma_{SL}$ were carried out for n=1 in the Gibbs isotherm equation. It was confirmed that the isotherms of $\Gamma_{SL}$ (Figure S29) are comparable to those obtained earlier for the W-A interface ($\Gamma_{LV}$) using the same assumption [32]. It is interesting that the maximal excess concentration of a given extract fraction ($\Gamma_{SL}^{max})$ is similar to that calculated based on Equation (7) (Table 3).

It should be emphasized that there is an agreement between the $\Gamma_{SL}$ obtained from the Gibbs isotherm equation [35] and that obtained based on the slope of the linear function between $\gamma_{LV}\cos\theta$ and $\gamma_{LV}$.

The excess concentration of the extract fraction at the PTFE,*L* interface can be calculated using the Frumkin equation [36]. The shape of the $\Gamma_{SL}$ isotherms obtained using the Frumkin equation is insignificantly different for the given extract fraction (Figure S29). However, it is similar to the isotherms of $\Gamma_{LV}$ [35], which confirms the conclusion that the adsorption of the extract fractions at the PTFE,*L* and L-A interfaces is comparable. This indicates that the tendency to adsorb the extract fraction at both these interfaces is similar, which can be confirmed based on the standard Gibbs free energy of adsorption ($\Delta G_{ads}^{0}$). Since the isotherms of $\Gamma_{SL}$ for the extract fraction are known, it is possible to determine $\Delta G_{ads}^{0}$ from the Langmuir equation modified by de Boer [35,37]. As the extract fraction includes the substances characterized by molar weight in the range from 500 to 3000 g, these values were applied for the *C* calculation [35]. Thus, two values of $\Delta G_{ads}^{0}$ for each extract fraction obtained from the modified Langmuir equation (Table 3) are similar to those obtained for the L-A interface [35]. This points out that the tendency of the extract fractions to adsorb at the PTFE,*L* and L-A interfaces is the same. The $\Delta G_{ads}^{0}$ values can be also obtained using the *a* constant in the Szyszkowski equation, which fulfils the following equation [12]:

(8) $$a=\omega \exp\frac{\Delta G_{ads}^{0}}{RT},$$

where in this case, $a=\frac{a_{1}}{M}$.

The values of $\Delta G_{ads}^{0}$ calculated using Equation (8) based on the constant $a_{1}$ in Equation (7) are similar to those determined from the modified Langmuir equation and to the $\Delta G_{ads}^{0}$ values obtained for the solution–air interface (Table 3).

The determination of the extract fraction excess concentration at the PMMA,*L* and G-L interfaces is more complex than that at the PTFE,*L* interface. As confirmed above in the case of the PMMA (glass)–solution drop–air system, the surface tension of PMMA and/or glass depends on $C_{E}$. Thus $\Gamma_{SV}>0$, and based on the linear dependence between $\gamma_{LV}\cos\theta$ and $\gamma_{LV}$, it is impossible to deduce the $\Gamma_{SL}$ values. At $\Gamma_{SV}>0$, the slope of this dependence is different from −1, and in the case of glass, even becomes positive. If $\Gamma_{SV}>0$, the positive slope of the linear dependence between $\gamma_{LV}\cos\theta$ and $\gamma_{LV}$ confirms that the adsorption of substrates at the S-L interface is not negative, but that $\Gamma_{SV}>\Gamma_{SL}$ [33].

$\Gamma_{SV}$ and $\Gamma_{SL}$ were calculated based on the suitable form of Gibbs isotherm equation [34]. The values of $\gamma_{SV}$ used for the calculation of $\Gamma_{SV}$ were established as equal to PMMA or glass surface tension minus π. The π values were obtained based on the difference between $W_{a}$ for PMMA or the glass surface calculated using van Oss et al. [18,19,20] and EY-D [9]. After application of the $\gamma_{SV}$ values determined in such way in the Young equation, the $\gamma_{SL}$ values were obtained and used for the determination of $\Gamma_{SL}$. The values of $\Gamma_{SV}$ and $\Gamma_{SL}$ were also obtained using the Frumkin equation (Figures S30–S33). The shape of the $\Gamma_{SV}$ and $\Gamma_{SL}$ isotherms calculated based on the Frumkin and Gibbs equations is slightly different (Figures S30–S33). However, based on the $\Gamma_{SV}$ and $\Gamma_{SL}$ calculations using two methods, the adsorption of the extract fractions at the PMMA,*L* and G-L interfaces is considerably lower than that at the L-A interface (Figures S30–S33). It depends largely on the type of extract fraction. For PMMA $\Gamma_{SV}$ < $\Gamma_{SL}$. However, for glass $\Gamma_{SV}$ > $\Gamma_{SL}$ in the case of E2, E3 and E4. This may result from the fact that there are repulsive electrostatic interactions at the G-L interface between the negative charge on the glass surface and the negative COO^-^ ions. It should be mentioned that the orientation of the extract fraction molecules at the PMMA,*L*, G-L and L-A interfaces may be different. The tendency to adsorb the extract fraction at three interfaces is different, which is confirmed by $\Delta G_{ads}^{0}$ (Table 3). It should also be mentioned that for PMMA and glass (except for E1), ($\Gamma_{SV}-\;\Gamma_{SL}$)/$\Gamma_{LV}$ [30] is close for the slope of the linear dependence between $\gamma_{LV}\cos\theta$ and $\gamma_{LV}$ (Table 3).

## 3. Materials and Methods

Fractions of *Saponaria officinalis* L. (Flos Sp. Z o.o., Pasłęk, Poland) saponins were obtained based on a previously described procedure [35].

Contact angle measurements were carried out on the glass surface with the use of formamide (>99.5%) and diiodomethane (>99%), which were bought from Sigma-Aldrich (Poland).

The PTFE and PMMA plates were obtained from Mega-Tech, Tomaszów Mazowiecki, Poland, while microscopic glasses (25 × 75 mm) (Poland) were used as glass plates. Prior to contact angle measurements, the PTFE, PMMA and glass plates were prepared in accordance with the previously described procedure [21].

The sessile drop method was used to determine the advancing contact angle for the aqueous solutions of the saponin extract fraction on the PTFE, PMMA and glass. Subsequent determinations were conducted using water, formamide and diiodomethane on the glass surface. The measurements were carried out with the use of the DSA30 measuring system (Krüss, Hamburg, Germany), in a thermostated chamber at 293 ± 0.1 K. In order to ensure proper reproducibility, the contact angle was measured for at least 30 drops of the given liquid or solution. The general standard deviation for each set of values did not exceed ±1.5°. Microsoft Excel 2013 software was used to conduct the statistical analysis.

In case of all experiments and measurements, a triplicate repetition was used. Arithmetic means and standard deviations were used in case of calculations. The calculations were conducted using the MATLAB software (MATLAB Online, The MathWorks, Natick, MA, USA)

## 4. Conclusions

The collected results elucidate the wetting properties of the different fractions of the extract isolated from *S. officinalis* roots. It was observed that the isotherms of the contact angle for PTFE, PMMA and glass can be described by the exponential function of the second order. In the case of PTFE, these isotherms can be also described by the modified Szyszkowski equation.

Moreover, for PTFE, there is a linear dependence between the adhesion and the surface tension, the slope of which is equal to −1. It does not depend on the type of extract fraction. The dependence between the cosine of contact and the surface tension of solution for PTFE is not linear. However, it can be described by one curve for all types of extract concentration. In the case of PTFE, there is a single linear dependence for all extract fractions between the contact angle and the logarithm of the sum of solution–air and PTFE–solution interface tension.

For PMMA and glass, the relations between the adhesion and the surface tension, as well as between the cosine of contact angle and the surface tension, depend on the type of extract fraction; however, all types of the extract can be described by the relationship between the contact angle and the logarithm of the sum of the solid–air, solid–solution and solution–air interfaces tension with one curve.

What is interesting is that there are differences between the values of adhesion of the aqueous solution of the extract fraction to the PMMA and the glass surface calculated based on the van Oss et al. and Young–Dupre equations, which probably result from the penetration of some substrates from the solution drop settled on the PMMA or glass surface and decrease their surface tension.

The dependence between the adhesion calculated using the van Oss et al. equation and the surface tension of the solution can be described by a single linear function for all types of extract fractions. For all tested materials, the purified fraction of the extract presented better surface activity, which confirms the rationale of extract purification via membrane separation. Additionally, the collected data show that *S. officinalis* is a promising source of natural surface active compounds, which can be used as a “green” wetting agent characterized by properties comparable with synthetic surfactants.

## Figures and Tables

**Table 1:** The values of the constants in the exponential function of the second order for PTFE, PMMA and glass.

Fraction	$y_{0}$	$A_{1}$	$t_{1}$	$A_{2}$	$t_{2}$
**PTFE**					
E0	89.84536	5.34745	0.05219	15.26601	0.77681
E1	87.70078	3.12596	0.1369	19.76883	1.49907
E2	89.34443	5.57735	0.05371	15.71605	0.82901
E3	84.94747	19.48124	2.7536	5.87263	0.08604
E4	92.02671	6.71405	0.06221	9.44685	0.39655
**PMMA**					
E0	42.63048	24.11468	0.90393	6.75639	0.05314
**Glass**					
E0	21.62536404	28.02520023	0.83668873	6.0955882	0.05097999

**Table 2:** The values of $\gamma_{C}$ determined from the dependence between adhesion and surface tension of solution.

Fraction	Slope	Constant	$\gamma_{C}\;$ [mN/m]
**PTFE**			
E0	−0.99927	46.54963	23.28331
E1	−0.99741	46.47121	23.26573
E2	−0.99696	46.43604	23.25337
E3	−1.00392	46.86771	23.38801
E4	−1.00165	46.71216	23.33683
**PMMA**			
E0	−0.53197	58.50527	38.18957
E1	−0.55895	60.50714	38.81275
E2	−0.47278	54.28383	36.85807
E3	−0.43314	51.2961	35.7928
E4	−0.37529	47.19545	34.31673
**Glass**			
E0	−0.15585353	51.39862153	60.88827
E1	-	-	-
E2	0.06059295	35.62625951	33.59089
E3	0.13605857	30.06522735	26.4645
E4	0.14747917	29.23123737	25.47431
E0	−0.15585353	51.39862153	60.88827

**Table 3:** The values of maximal Gibbs surface excess concentration ($\Gamma^{max}$) calculated for PTFE, PMMA and glass from the Gibbs isotherm equation, as well as applied in Equation (7). The values of the standard Gibbs free energy of adsorption ($\Delta G_{ads}^{0}$ ) calculated from the modified Langmuir equation and Equation (8).

Fraction	$\Gamma^{max}$ (⨯10^−6^ mol/m^2^)				$∆G_{ads}^{0}$ (kJ/mol) from Modified Langmuir Equation [32,34]				$∆G_{ads}^{0}$(kJ/mol) Equation (8)		
	from Gibbs Equation [31]			Equation (7)	500		3000		500		3000
**PTFE**											
E0	1.93			1.8	−32.67		−37.06		−34.07	−38.43	
E1	2.30			2.4	−29.42		−33.74		−30.56	−34.93	
E2	1.93			1.9	−32.83		37.20		−33.45	−37.82	
E3	1.70			1.7	−30.83		−35.24		−33.06	−37.42	
E4	1.81			1.8	−32.23		−36.59		−36.17	−40.54	
**PMMA**											
	$\Gamma^{max}$ (⨯10^−6^ mol/m^2^)		$\frac{\Gamma_{SV}-\Gamma_{SL}}{\Gamma_{LV}}$	$\Gamma^{max}$ Equation (7)	$\Delta G_{ads}^{0}$ (kJ/mol) from modified Langmuir equation [32,34]				$\Delta G_{ads}^{0}$ (kJ/mol) Equation (8)		
	SV	SL									
	from Gibbs equation [31]										
					500		3000		500	3000	
					SV	SL	SV	SL			
E0	0.24	1.25	−0.52	1.0	−26.82	−31.28	−31.18	−35.54	−35.46	−39.83	
E1	0.25	1.61	−0.51	-	−24.22	−28.33	−28.51	−32.65	-	-	
E2	0.36	1.27	−0.45	-	−27.72	−31.33	−32.53	−35.60	-	-	
E3	0.39	1.09	−0.42	-	−27.36	−30.04	−31.62	−34.38	-	-	
E4	0.49	1.19	−0.39	-	−28.76	−31.13	−33.13	−35.42	-	-	
**Glass**											
	$\Gamma^{max}$ (⨯10^−6^ mol/m^2^)		$\frac{\Gamma_{SV}-\Gamma_{SL}}{\Gamma_{LV}}$	$\Gamma^{max}$ Equation (7)	$\Delta G_{ads}^{0}$ (kJ/mol) from modified Langmuir equation [32,34]				$\Delta G_{ads}^{0}$ (kJ/mol) Equation (8)		
	SV Equation	SL Equation			500		3000		500	3000	
	from Gibbs equation [31]				SV	SL	SV	SL			
E0	0.38	0.68	−0.15	1.4	−28.61	−29.65	−32.97	−33.81	−36.78	−41.15	
E1	1.13	0.87	-	-	−22.75	−27.04	−27.11	−31.35	-	-	
E2	0.78	0.69	0.066	-	−30.33	−29.68	−34.57	−33.85	-	-	
E3	0.83	0.60	0.13	-	−29.33	−28.48	−33.69	−32.85	-	-	
E4	0.82	0.58	0.14	-	−30.06	−29.37	−34.43	−33.60	-	-	

## Data Availability

The data presented in this study are available in Supplementary Materials.

## Supplementary Materials

The following supporting information can be downloaded at: https://www.mdpi.com/article/10.3390/molecules28134943/s1, Figure S1: A plot of the contact angle (θ) measured on the PTFE surface vs. the logarithm from the fraction concentration (log m). Figure S2: A plot of the contact angle (θ) measured on the PTFE surface vs. the aqueous solution surface tension. Figure S3: A plot of the contact angle (θ) measured for the aqueous solution of fraction E0 on the PTFE surface (points 1) and calculated from Equation (7) (curve 2), Equation (6) (curve 3) and expression $\cos\theta =-1+\frac{W_{a}}{\gamma_{LV}}$ vs. the logarithm from the fraction concentration (log m). Figure S4: A plot of the contact angle (θ) measured for the aqueous solution of fraction E1 on the PTFE surface (points 1) and calculated from Equation (7) (curve 2), Equation (6) (curve 3) and expression $\cos\theta =-1+\frac{W_{a}}{\gamma_{LV}}$ vs. the logarithm from the fraction concentration (log m). Figure S5: A plot of the contact angle (θ) measured for the aqueous solution of fraction E2 on the PTFE surface (points 1) and calculated from Equation (7) (curve 2), Equation (6) (curve 3) and expression $\cos\theta =-1+\frac{W_{a}}{\gamma_{LV}}$ vs. the logarithm from the fraction concentration (log m). Figure S6: A plot of the contact angle (θ) measured for the aqueous solution of fraction E3 on the PTFE surface (points 1) and calculated from Equation (7) (curve 2), Equation (6) (curve 3) and expression $\cos\theta =-1+\frac{W_{a}}{\gamma_{LV}}$ vs. the logarithm from the fraction concentration (log m). Figure S7: A plot of the contact angle (θ) measured for the aqueous solution of fraction E4 on the PTFE surface (points 1) and calculated from Equation (7) (curve 2), Equation (6) (curve 3) and expression $\cos\theta =-1+\frac{W_{a}}{\gamma_{LV}}$ vs. the logarithm from the fraction concentration (log m). Figure S8: A plot of the contact angle (θ) measured on the PTFE surface vs. the logarithm of the sum of the solution surface tension and solid–liquid interface tension ($\gamma_{LV}+\gamma_{PTFE,L}$). Figure S9: A plot of the adhesion tension ($\gamma_{LV}\cos\theta$) vs. the aqueous solution surface tension ($\gamma_{LV}$) for PTFE. Figure S10: A plot of the cosine of the contact angle vs. the aqueous solution surface tension ($\gamma_{LV}$) for PTFE. Figure S11: A plot of the contact angle (θ) measured on the PMMA surface vs. the logarithm of the sum of the solution and PMMA surface tension and solid–liquid interface tension ($\gamma_{PMMA,A}+\gamma_{PMMA,L}+\gamma_{LV}$). Figure S12: A plot of the adhesion work ($W_{a}$) of the solution to the PMMA surface calculated from the expression $W_{a}=2\sqrt{\gamma_{LV}^{LW}\gamma_{SV}^{LW}}+2\sqrt{\gamma_{LV}^{+}\gamma_{SV}^{-}}$ (curves 1–5) and from $W_{a}=\gamma_{LV}\left(\cos\theta +1\right)$ (curves 1′–5′) vs. the solution surface tension ($\gamma_{LV}$). Figure S13: A plot of the contact angle (θ) measured for the aqueous solution of fraction E0 on the PMMA surface (points 1) and calculated from Equation (6) (curve 2) and Equation (7) (curve 3) vs. the logarithm from the fraction concentration (log m). Figure S14: A plot of the contact angle (θ) measured for the aqueous solution of fraction E1 on the PMMA surface (points 1) and calculated from Equation (6) (curve 2) vs. the logarithm from the fraction concentration (log m). Figure S15: A plot of the contact angle (θ) measured for the aqueous solution of fraction E2 on the PMMA surface (points 1) and calculated from Equation (6) (curve 2) vs. the logarithm from the fraction concentration (log m). Figure S16: A plot of the contact angle (θ) measured for the aqueous solution of fraction E3 on the PMMA surface (points 1) and calculated from Equation (6) (curve 2) vs. the logarithm from the fraction concentration (log m). Figure S17: A plot of the contact angle (θ) measured for the aqueous solution of fraction E4 on the PMMA surface (points 1) and calculated from Equation (6) (curve 2) vs. the logarithm from the fraction concentration (log m). Figure S18: A plot of the adhesion tension ($\gamma_{LV}\cos\theta$) vs. the aqueous solution surface tension ($\gamma_{LV}$) for PMMA. Figure S19: A plot of the cosine of the contact angle vs. the aqueous solution surface tension ($\gamma_{LV}$) for PMMA. Figure S20: A plot of the contact angle (θ) measured for the aqueous solution of fraction E0 on the glass surface (points 1) and calculated from Equation (6) (curve 2) and Equation (7) (curve 3) vs. the logarithm from the fraction concentration (log m). Figure S21: A plot of the contact angle (θ) measured for the aqueous solution of fraction E1 on the glass surface (points 1) and calculated from Equation (6) (curve 2) vs. the logarithm from the fraction concentration (log m). Figure S22: A plot of the contact angle (θ) measured for the aqueous solution of fraction E2 on the glass surface (points 1) and calculated from Equation (6) (curve 2) vs. the logarithm from the fraction concentration (log m). Figure S23: A plot of the contact angle (θ) measured for the aqueous solution of fraction E3 on the glass surface (points 1) and calculated from Equation (6) (curve 2) vs. the logarithm from the fraction concentration (log m). Figure S24: A plot of the contact angle (θ) measured for the aqueous solution of fraction E4 on the glass surface (points 1) and calculated from Equation (6) (curve 2) vs. the logarithm from the fraction concentration (log m). Figure S25: A plot of the adhesion tension ($\gamma_{LV}\cos\theta$) vs. the aqueous solution surface tension ($\gamma_{LV}$) for glass. Figure S26: A plot of the cosine of the contact angle vs. the aqueous solution surface tension ($\gamma_{LV}$) for PMMA. Figure S27: A plot of the adhesion work ($W_{a}$) of the solution to the glass surface calculated from the expression $W_{a}=2\sqrt{\gamma_{LV}^{LW}\gamma_{SV}^{LW}}+2\sqrt{\gamma_{LV}^{+}\gamma_{SV}^{-}}\;$(curves 1–5) and from $W_{a}=\gamma_{LV}\left(\cos\theta +1\right)$ (curves 1′–5′) vs. the solution surface tension ($\gamma_{LV}$). Figure S28: A plot of the contact angle (θ) measured on the glass surface vs. the logarithm of the sum of the solution and PMMA surface tension and solid–liquid interface tension ($\gamma_{LV}+\gamma_{G,A}+\gamma_{G,L}$). Figure S29: A plot of the surface concentration at the PTFE–solution interface ($\Gamma_{SL}$) calculated from the Gibbs (curves 1–5) and modified Langmuir equations (11) (curves 1′–5′) vs. the logarithm from the fraction concentration (log m). Figure S30: A plot of the surface concentration at the PMMA–solution interface ($\Gamma_{SL}$) calculated from the Gibbs (curves 1–5) and modified Langmuir equations (curves 1′–5′) vs. the logarithm from the fraction concentration (log m). Figure S31: A plot of the surface concentration at the PMMA–air interface ($\Gamma_{SV}$) calculated from the Gibbs (13) (curves 1–5) and modified Langmuir equations (curves 1′–5′) vs. the logarithm from the fraction concentration (log m). Figure S32: A plot of the surface concentration at the glass–solution interface ($\Gamma_{SL}$) calculated from the Gibbs (curves 1–5) and modified Langmuir equations (curves 1′–5′) vs. the logarithm from the fraction concentration (log m). Figure S33: A plot of the surface concentration at the glass–air interface ($\Gamma_{SV}$) calculated from the Gibbs (curves 1–5) and modified Langmuir equations (curves 1′–5′) vs. the logarithm from the fraction concentration (log m).

## Sample Availability

Samples of the compounds are not available from the authors.
