# Supplementary material for: Thermodynamic Consideration of the Solid Saponin Extract Drop–Air System
Source: Molecules. 2023 Jun 23;28(13):4943. doi: 10.3390/molecules28134943 (PMC10343909; doi:10.3390/molecules28134943)
Supplement: Supplementary file 1 [file molecules-28-04943-s001.zip › molecules-2421276-supplementary.pdf]

Supplementary Information

Thermodynamic consideration of the solid-saponin extract drop-air system

Adam Grzywaczyk[1], Wojciech Smułek[1], Ewa Kaczorek[1], Anna Zdziennicka[2] and Bronisław Jańczuk[2]*

[1]Institute of Chemical Technology and Engineering, Poznań University of Technology, Berdychowo 4, 60-965 Poznań, Poland; adam.grzywaczyk@doctorate.put.poznan.pl (AG); wojciech.smulek@put.poznan.pl (WS); ewa.kaczorek@put.poznan.pl (EK)

[2]Department of Interfacial Phenomena, Institute of Chemical Sciences, Faculty of Chemistry, Maria Curie-Skłodowska University in Lublin, Maria Curie-Skłodowska Sq. 3, 20-031 Lublin, Poland; anna.zdziennicka@mail.umcs.pl (A.Z), bronislaw.janczuk@mail.umcs.pl (BJ)

*Corresponding author:

e-mail: bronislaw.janczuk@mail.umcs.pl, tel. +48 (81) 5375649

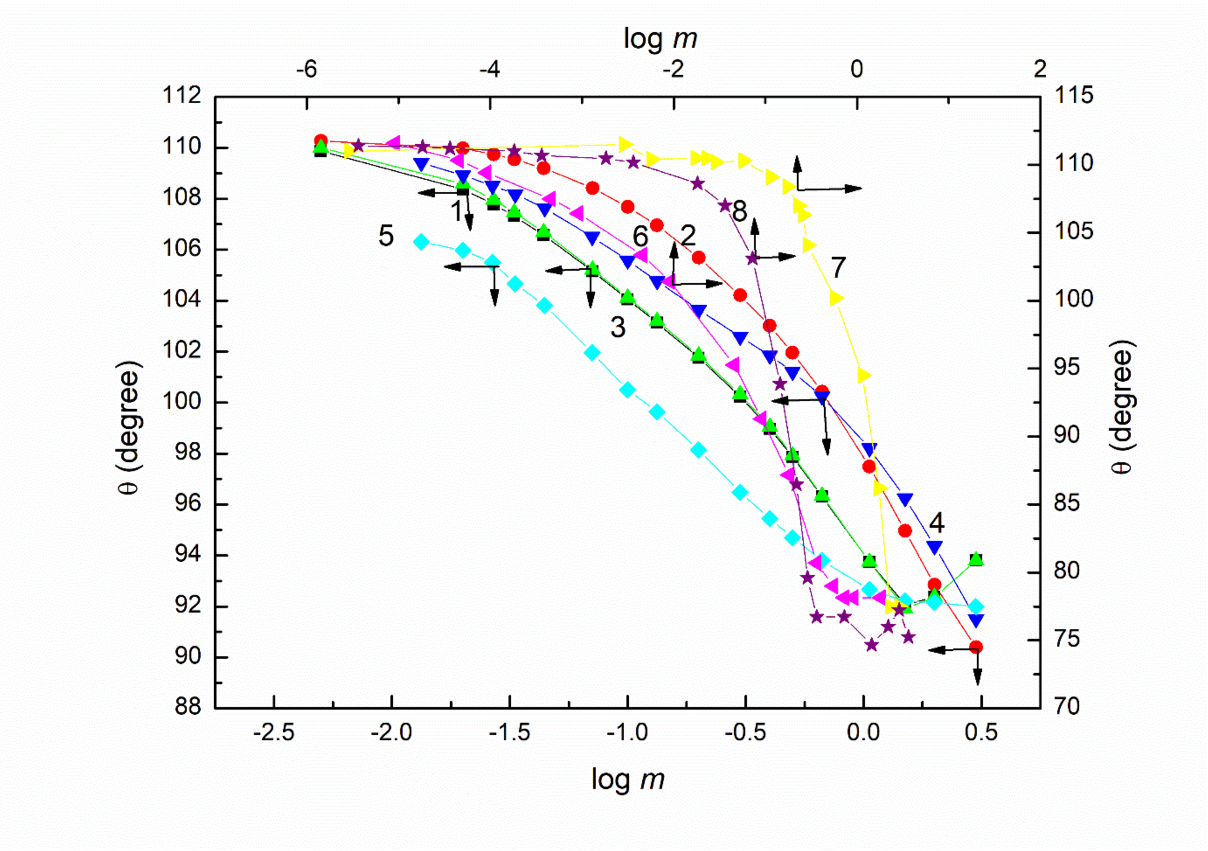

Figure S1. A plot of the contact angle (θ) measured on the PTFE surface vs. the logarithm of from the fraction concentration (log m). Curves 1 – 8 correspond to the fraction E0, E1, E2, E3, E4, TX165, SDS and CTAB respectively.

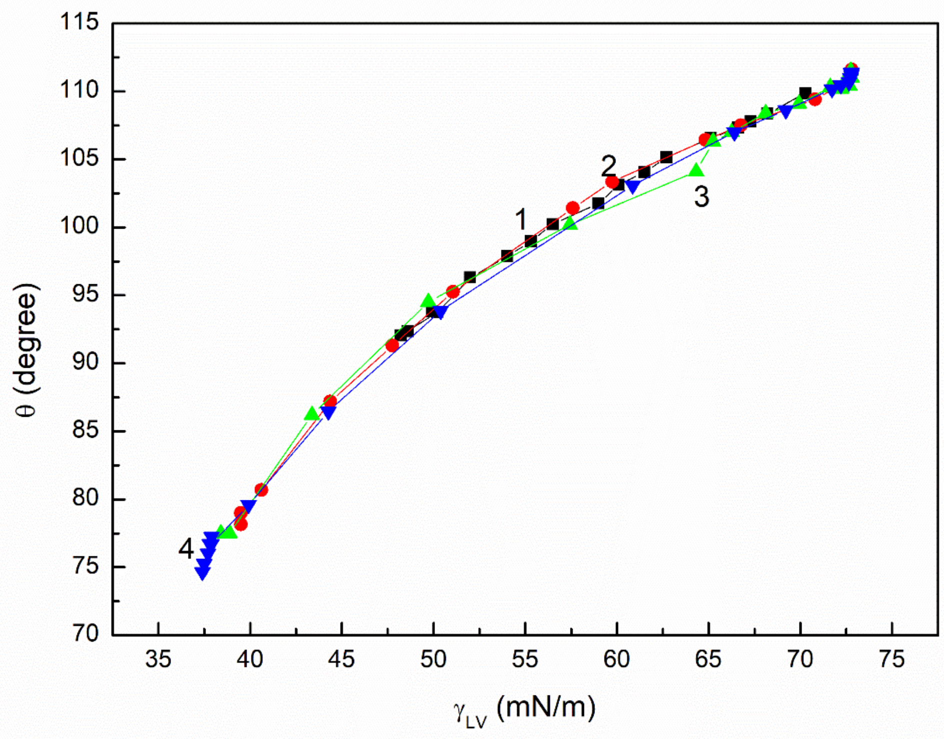

Figure S2. A plot of the contact angle (θ) measured on the PTFE surface vs. the aqueous solution surface tension. Curves 1 – 4 correspond to the fraction E0, TX165, SDS and CTAB respectively.

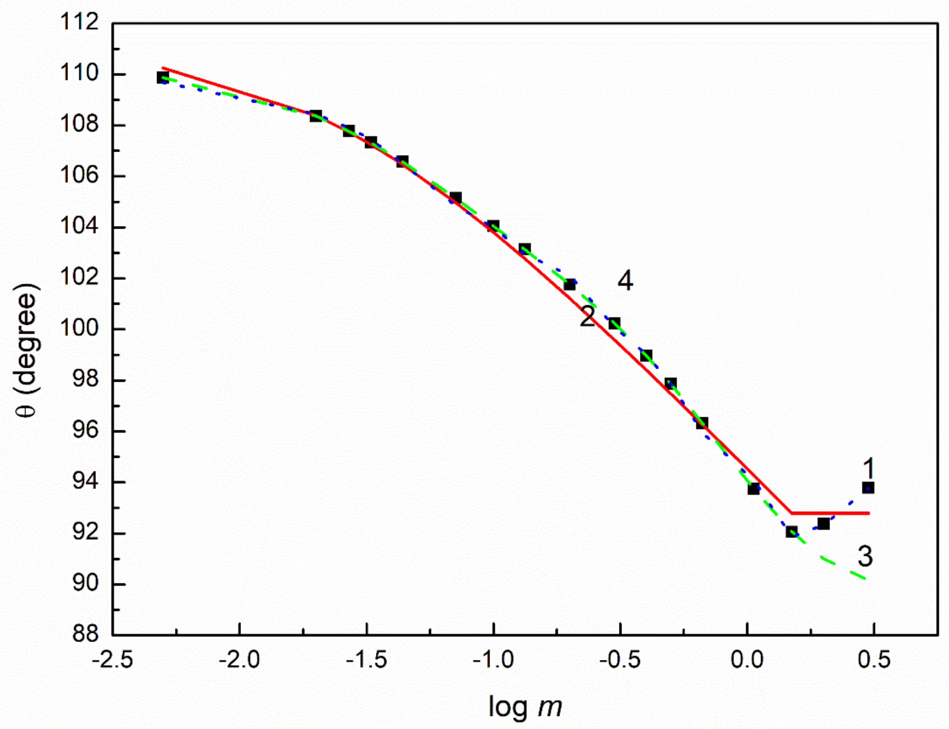

Figure S3. A plot of the contact angle (θ) measured for the aqueous solution of fraction E0 on the PTFE surface (points 1) and calculated from Equation (7) (curve 2), Equation (6) (curve 3) and from expression $\cos\theta = -1 + \frac{W_a}{\gamma_{LV}}$ vs. the logarithm from the fraction concentration ($\log m$).

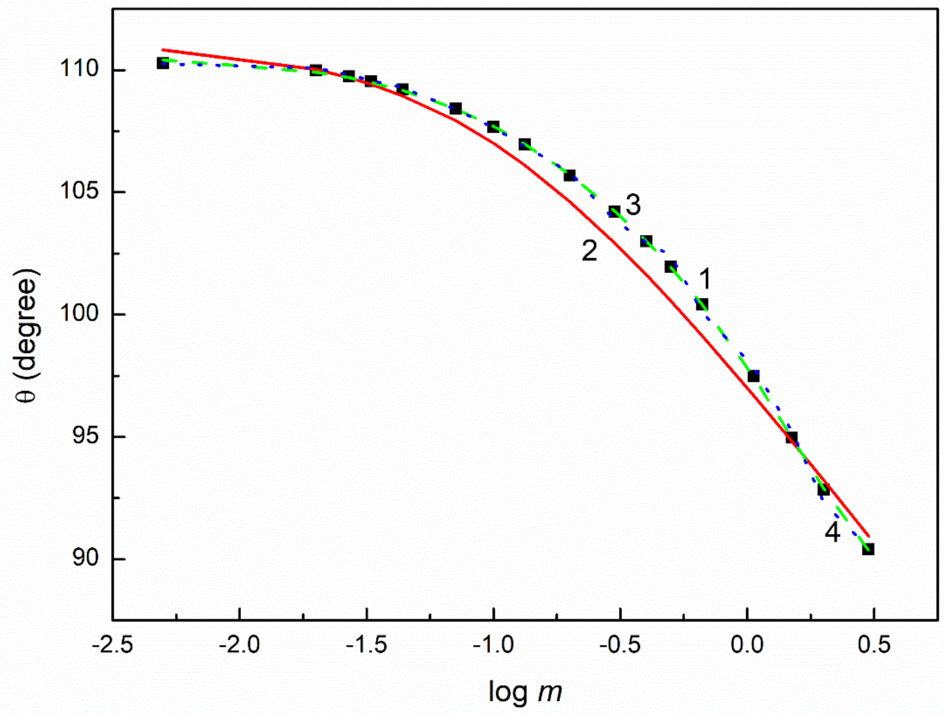

Figure S4. A plot of the contact angle (θ) measured for the aqueous solution of fraction E1 on the PTFE surface (points 1) and calculated from Equation (7) (curve 2), Equation (6) (curve 3) and from expression $\cos\theta = -1 + \frac{W_a}{\gamma_{LV}}$ vs. the logarithm from the fraction concentration ($\log m$).

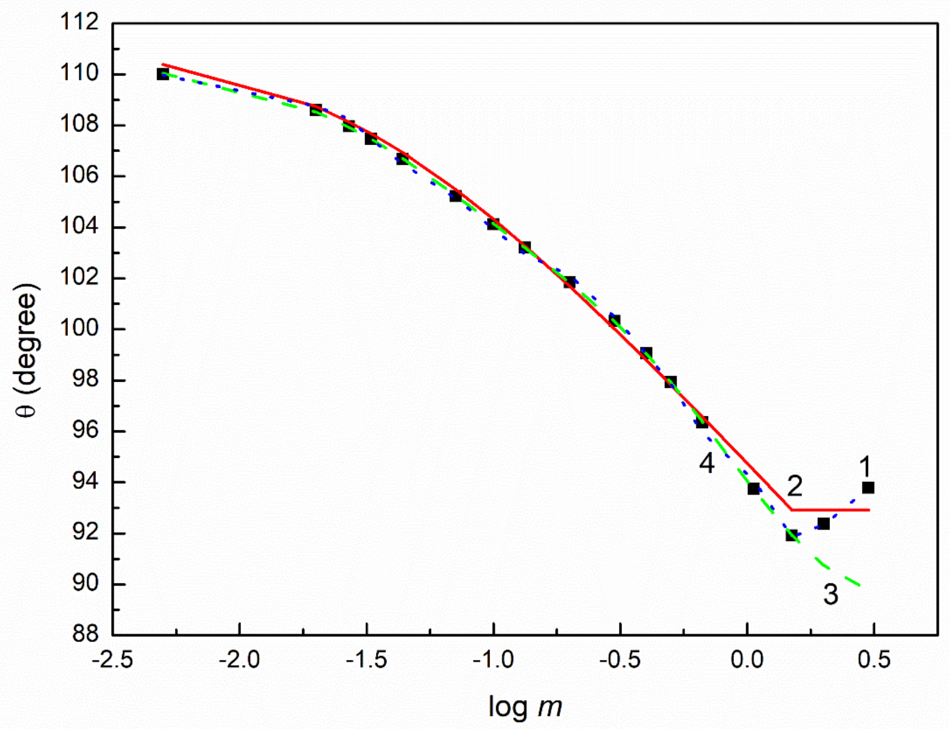

Figure S5. A plot of the contact angle (θ) measured for the aqueous solution of fraction E2 on the PTFE surface (points 1) and calculated from Equation (7) (curve 2), Equation (6) (curve 3) and from expression $\cos\theta = -1 + \frac{W_a}{\gamma_{LV}}$ vs. the logarithm from the fraction concentration ($\log m$).

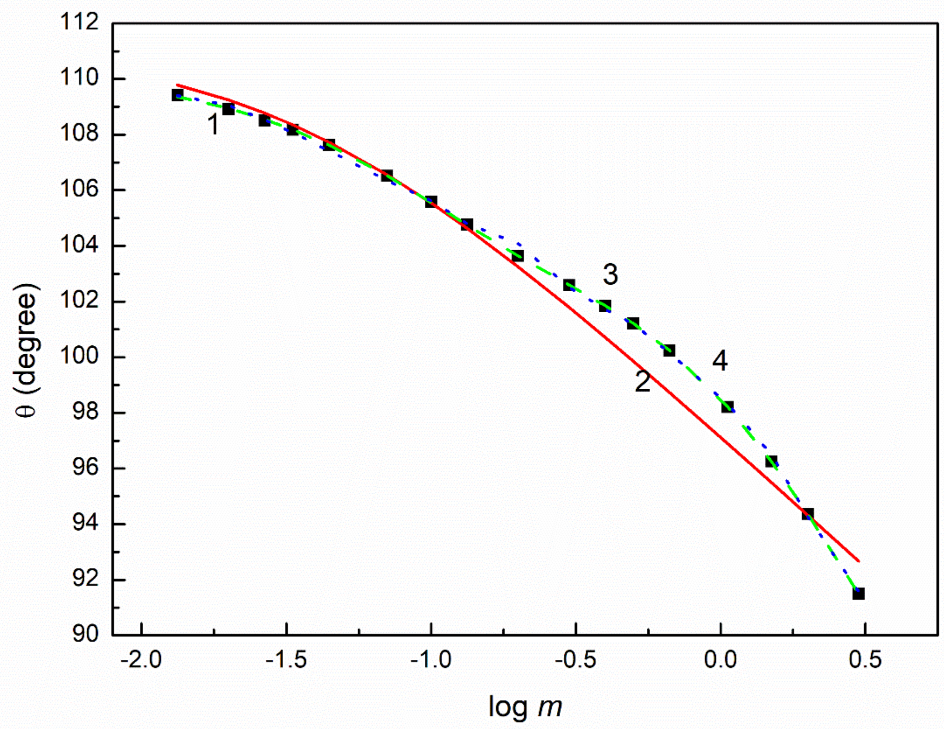

Figure S6. A plot of the contact angle (θ) measured for the aqueous solution of fraction E3 on the PTFE surface (points 1) and calculated from Equation (7) (curve 2), Equation (6) (curve 3) and from expression $\cos\theta = -1 + \frac{W_a}{\gamma_{LV}}$ vs. the logarithm from the fraction concentration ($\log m$).

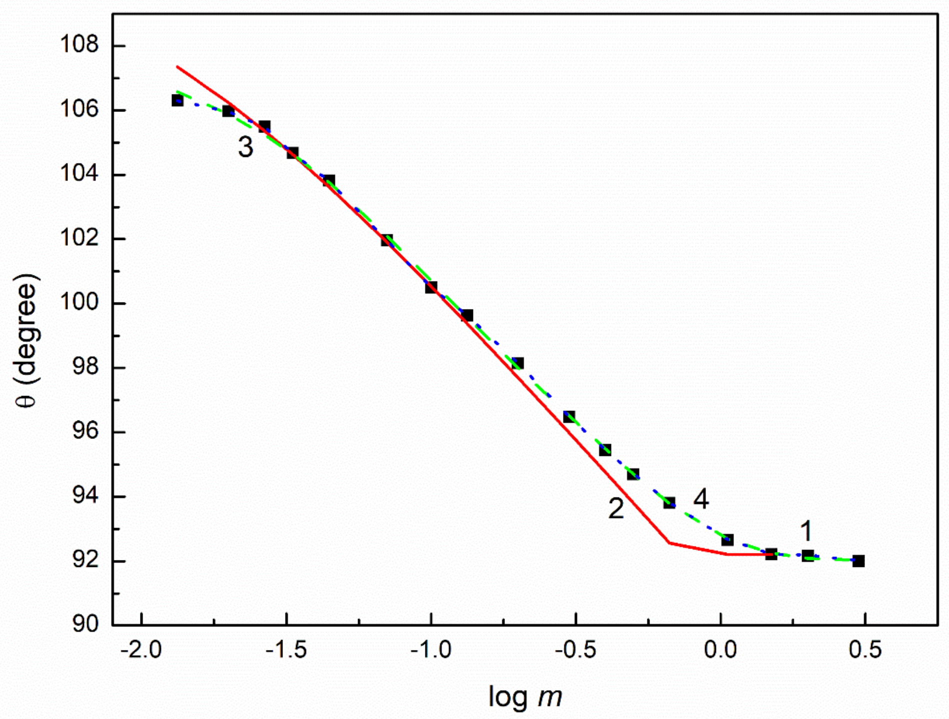

Figure S7. A plot of the contact angle (θ) measured for the aqueous solution of fraction E4 on the PTFE surface (points 1) and calculated from Equation (7) (curve 2), Equation (6) (curve 3) and from expression $\cos\theta = -1 + \frac{W_a}{\gamma_{LV}}$ vs. the logarithm from the fraction concentration ($\log m$).

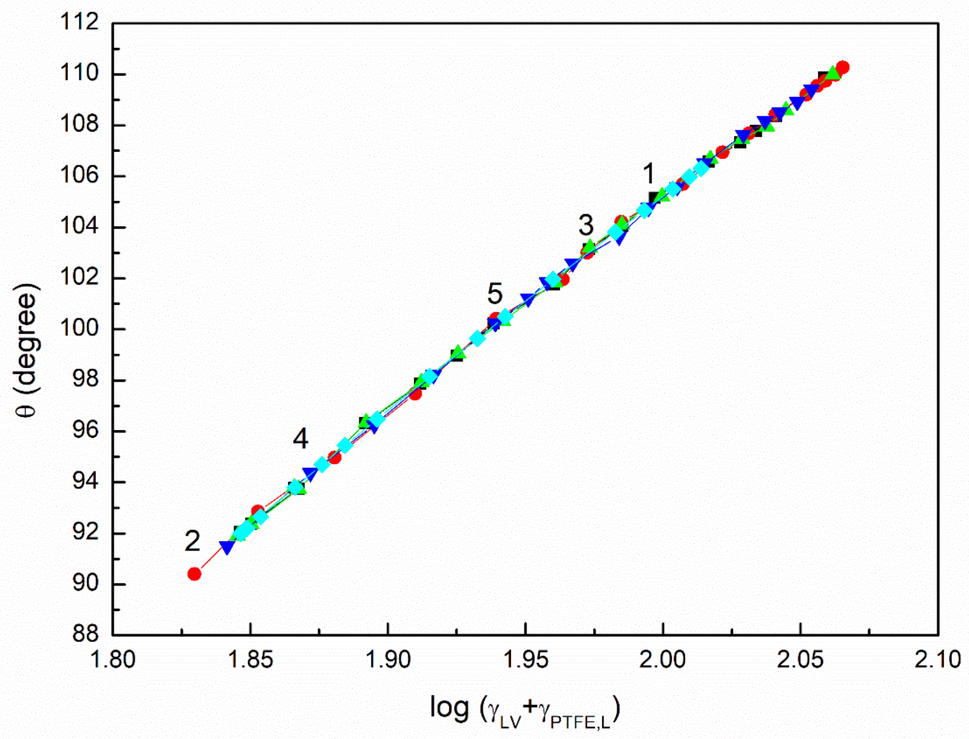

Figure S8. A plot of the contact angle (θ) measured on the PTFE surface vs. the logarithm of the sum of the solution surface tension and solid-liquid interface tension ($\gamma_{LV} + \gamma_{PTFE,L}$). Curves 1 – 5 correspond to the fraction E0, E1, E2, E3 and E4, respectively.

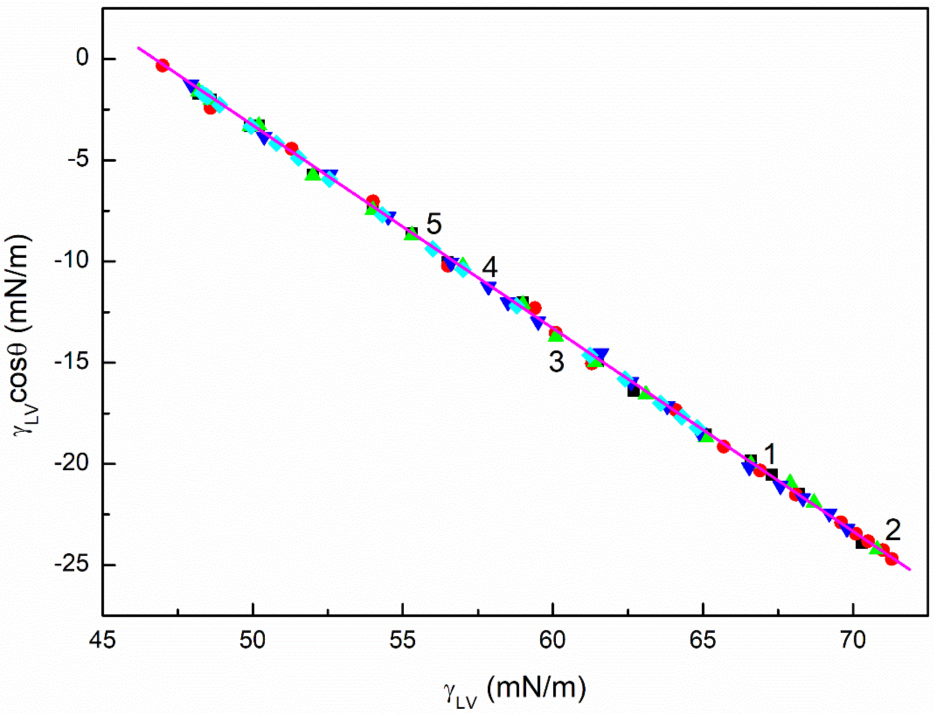

Figure S9. A plot of the adhesion tension ($\gamma_{LV}\cos\theta$) vs. the aqueous solution surface tension (γ_{LV}) for PTFE. Points 1 – 5 correspond to the fraction E0, E1, E2, E3 and E4, respectively.

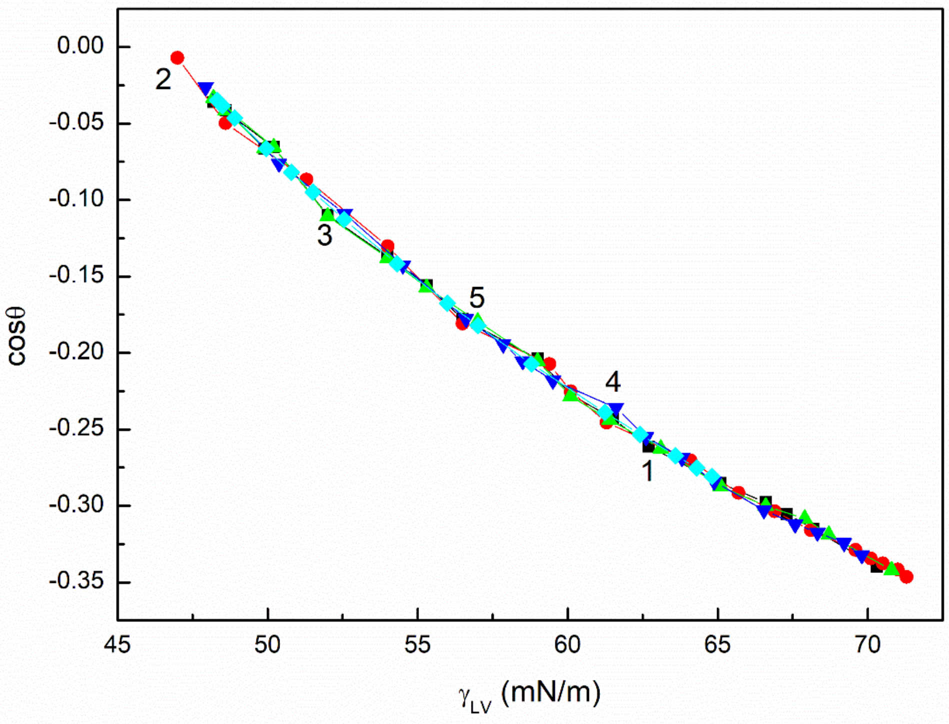

Figure S10. A plot of the cosine of the contact angle vs. the aqueous solution surface tension (γ_{LV}) for PTFE. Curves 1 – 5 correspond to the fraction E0, E1, E2, E3 and E4, respectively.

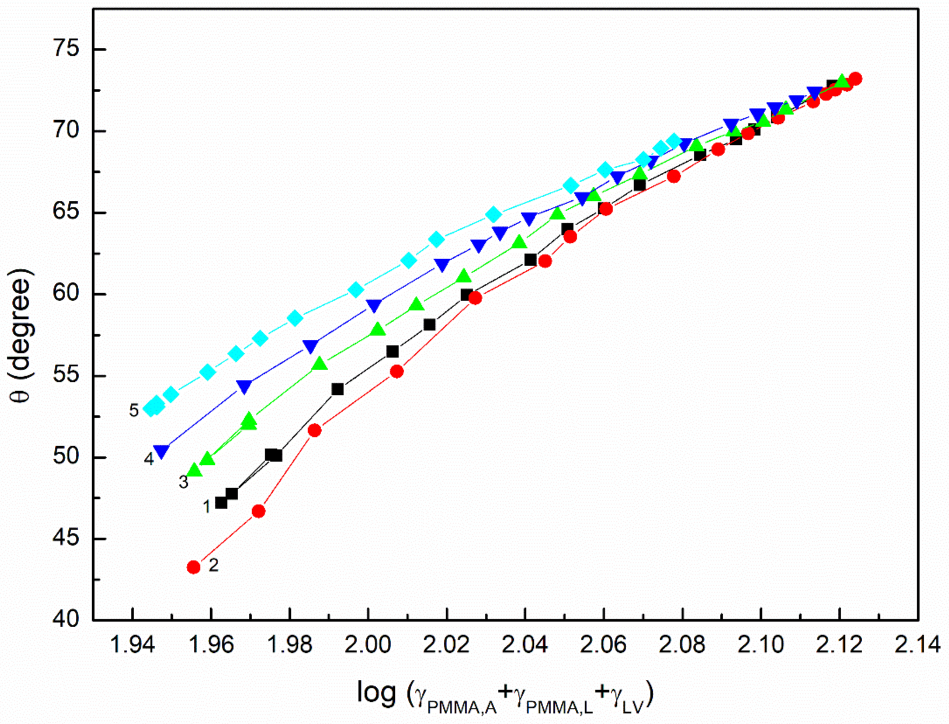

Figure S11. A plot of the contact angle (θ) measured on the PMMA surface vs. the logarithm of the sum of the solution and PMMA surface tension and solid-liquid interface tension ($\gamma_{PMMA,A} + \gamma_{PMMA,L} + \gamma_{LV}$). Curves 1 – 5 correspond to the fraction E0, E1, E2, E3 and E4, respectively.

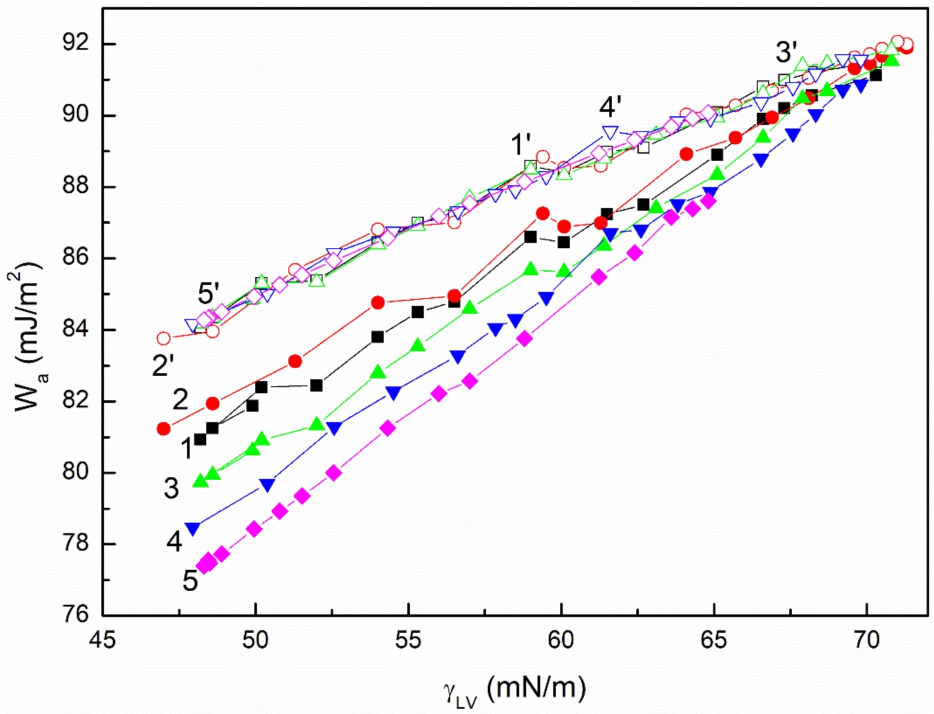

Figure S12. A plot of the adhesion work (W_a) of the solution to the PMMA surface calculated from the expression $W_a = 2\sqrt{\gamma_{LV}^{LW}\gamma_{SV}^{LW}} + 2\sqrt{\gamma_{LV}^{+}\gamma_{SV}^{-}}$ (curves 1 – 5) and from $W_a = \gamma_{LV}(\cos\theta + 1)$ (curves 1' – 5') vs. the solution surface tension (γ_{LV}). Curves 1 – 5 and 1' – 5'correspond to the fraction E0, E1, E2, E3 and E4, respectively.

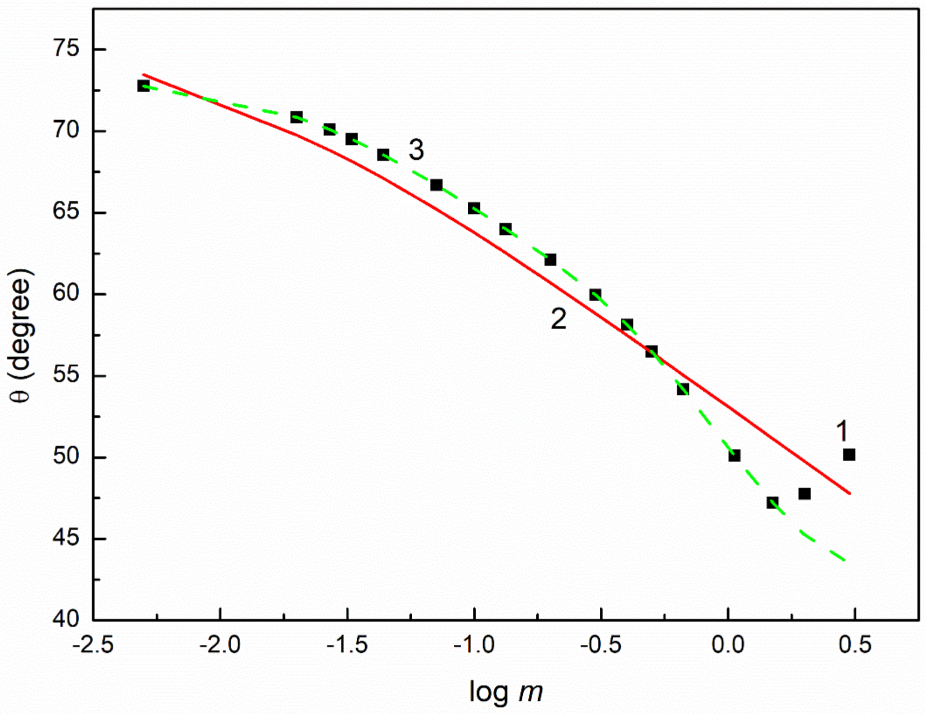

Figure S13. A plot of the contact angle (θ) measured for the aqueous solution of fraction E0 on the PMMA surface (points 1) and calculated from Equation (6) (curve 2) and Equation (7) (curve 3) vs. the logarithm from the fraction concentration (log m).

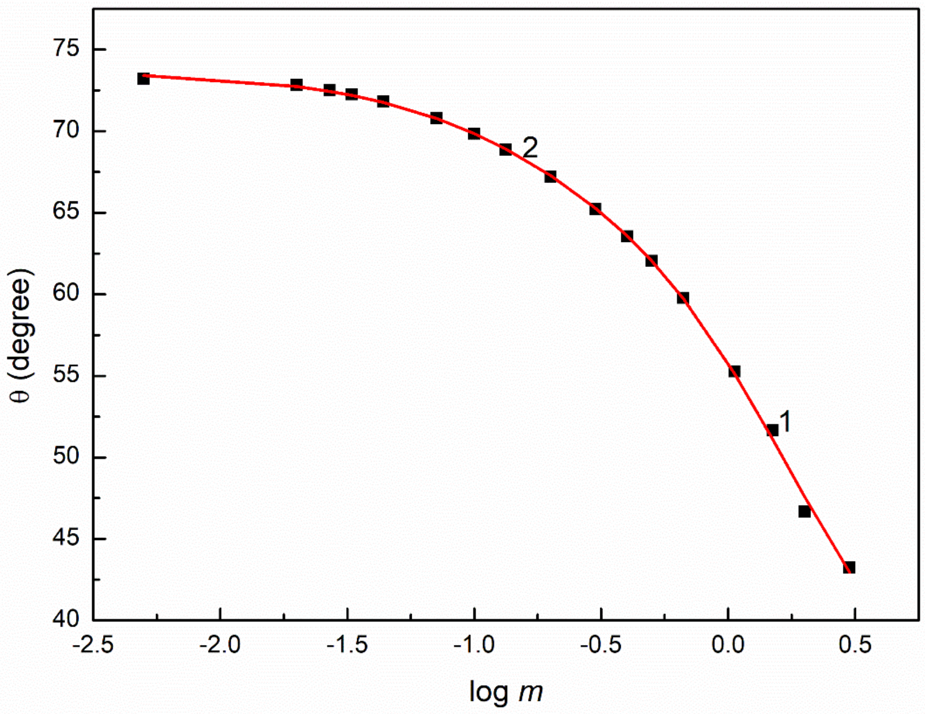

Figure S14. A plot of the contact angle (θ) measured for the aqueous solution of fraction E1 on the PMMA surface (points 1) and calculated from Equation (6) (curve 2) vs. the logarithm from the fraction concentration ($\log m$).

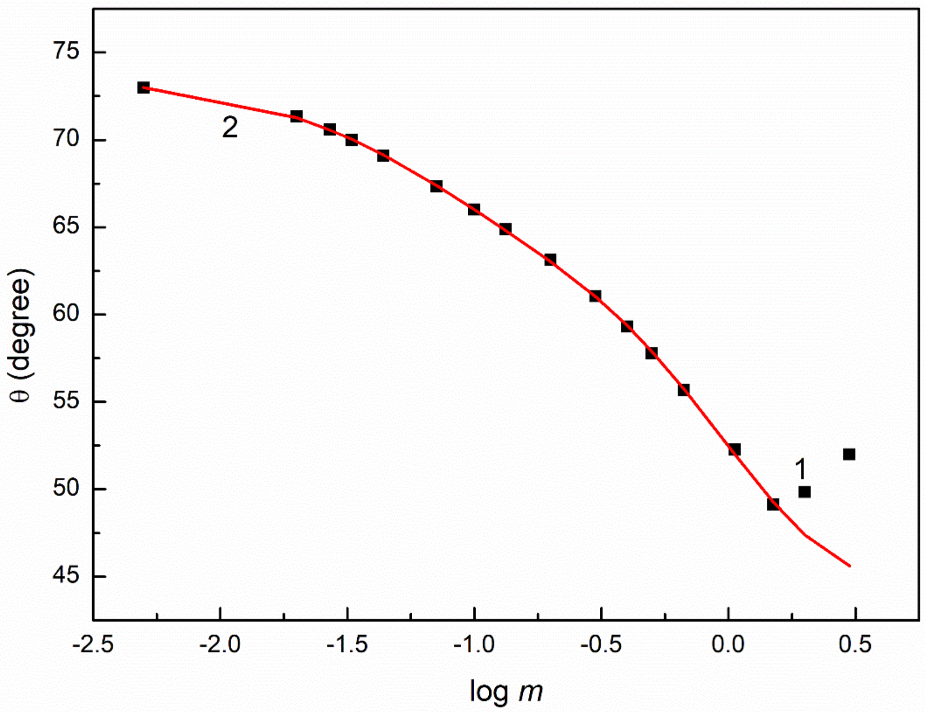

Figure S15. A plot of the contact angle (θ) measured for the aqueous solution of fraction E2 on the PMMA surface (points 1) and calculated from Equation (6) (curve 2) vs. the logarithm from the fraction concentration (log m).

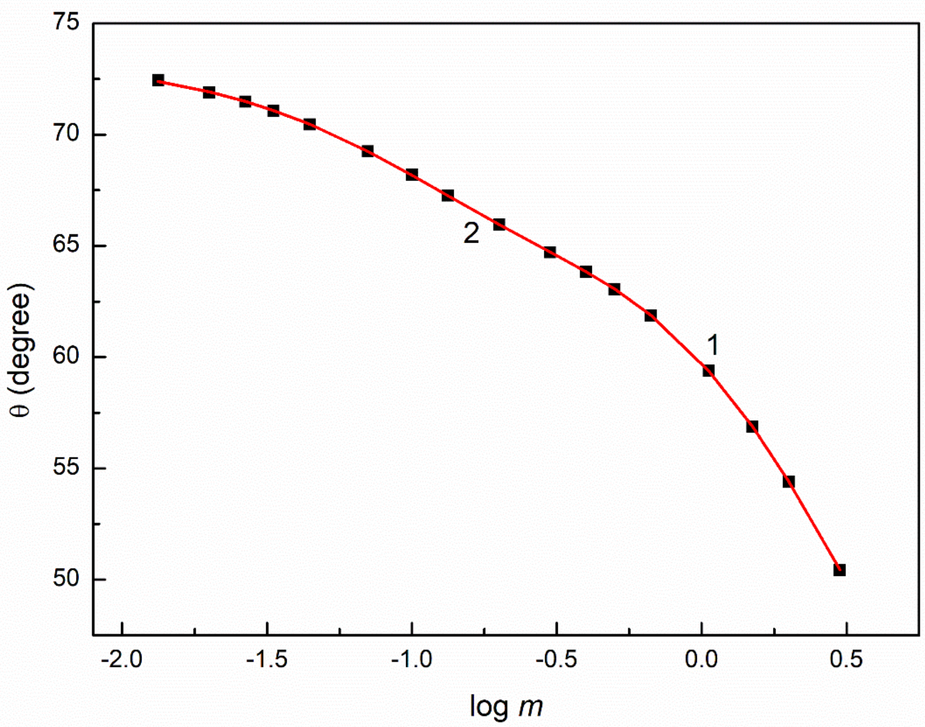

Figure S16. A plot of the contact angle (θ) measured for the aqueous solution of fraction E3 on the PMMA surface (points 1) and calculated from Equation (6) (curve 2) vs. the logarithm from the fraction concentration (log m).

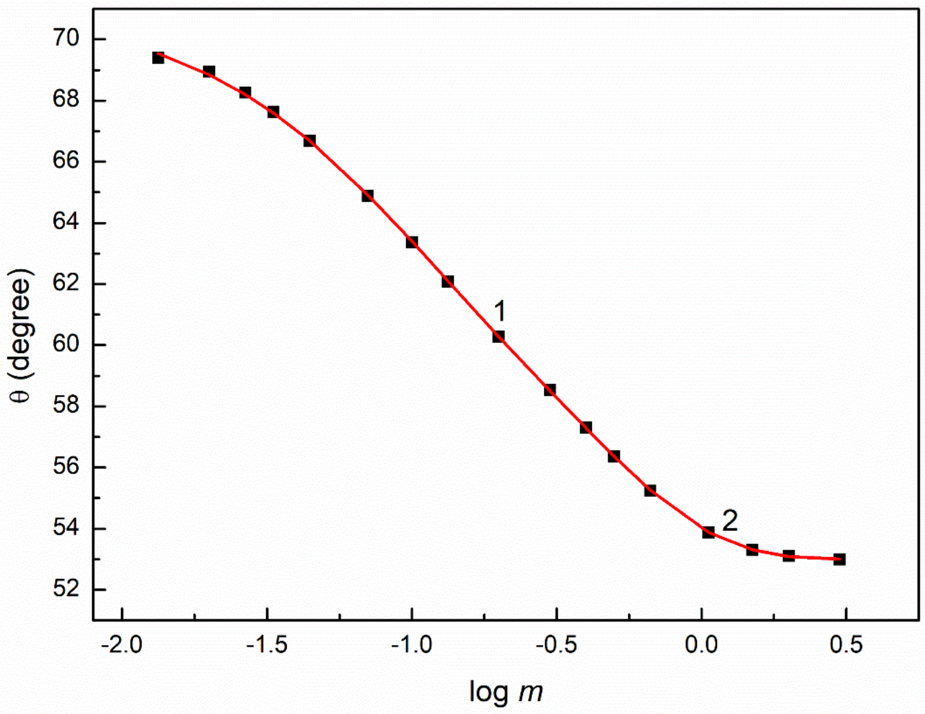

Figure S17. A plot of the contact angle (θ) measured for the aqueous solution of fraction E4 on the PMMA surface (points 1) and calculated from Equation (6) (curve 2) vs. the logarithm from the fraction concentration (log m).

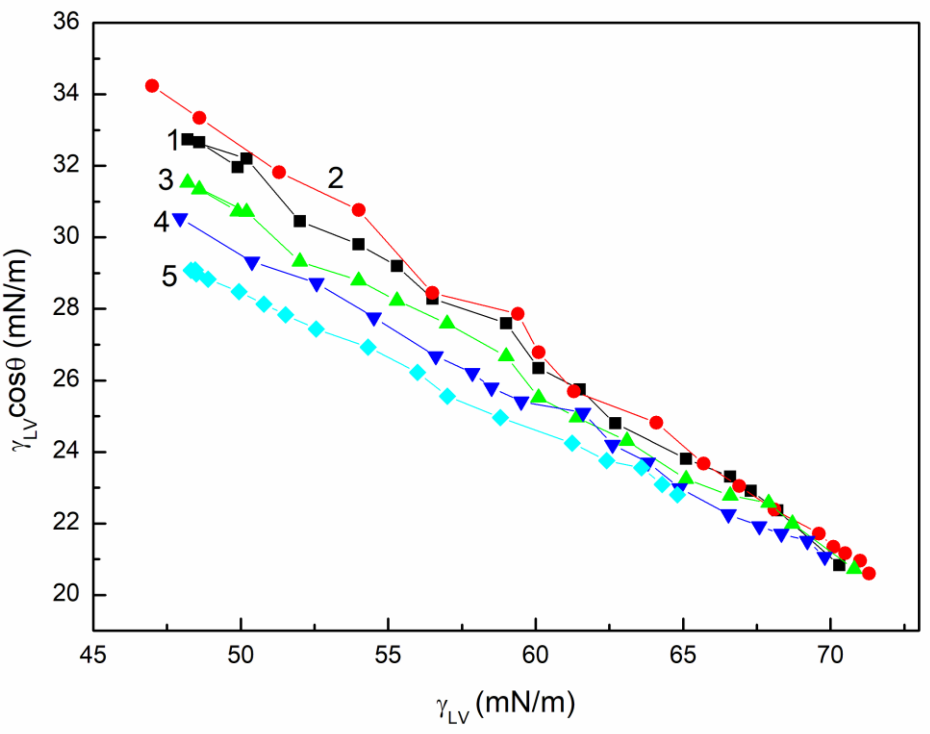

Figure S18. A plot of the adhesion tension ($\gamma_{LV}\cos\theta$) vs. the aqueous solution surface tension (γ_{LV}) for PMMA. Curves 1 – 5 correspond to the fraction E0, E1, E2, E3 and E4, respectively.

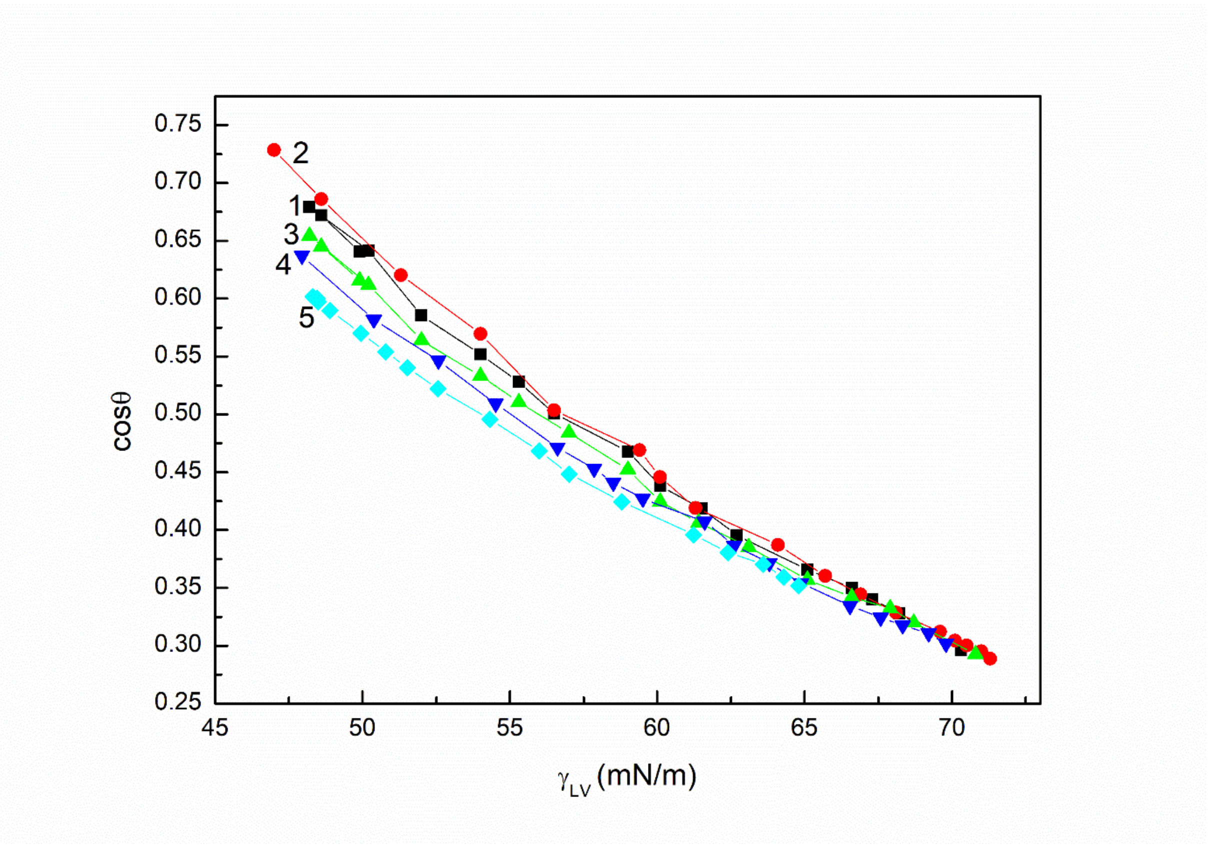

Figure S19. A plot of the cosine of the contact angle vs. the aqueous solution surface tension (γ_{LV}) for PMMA. Curves 1 – 5 correspond to the fraction E0, E1, E2, E3 and E4, respectively.

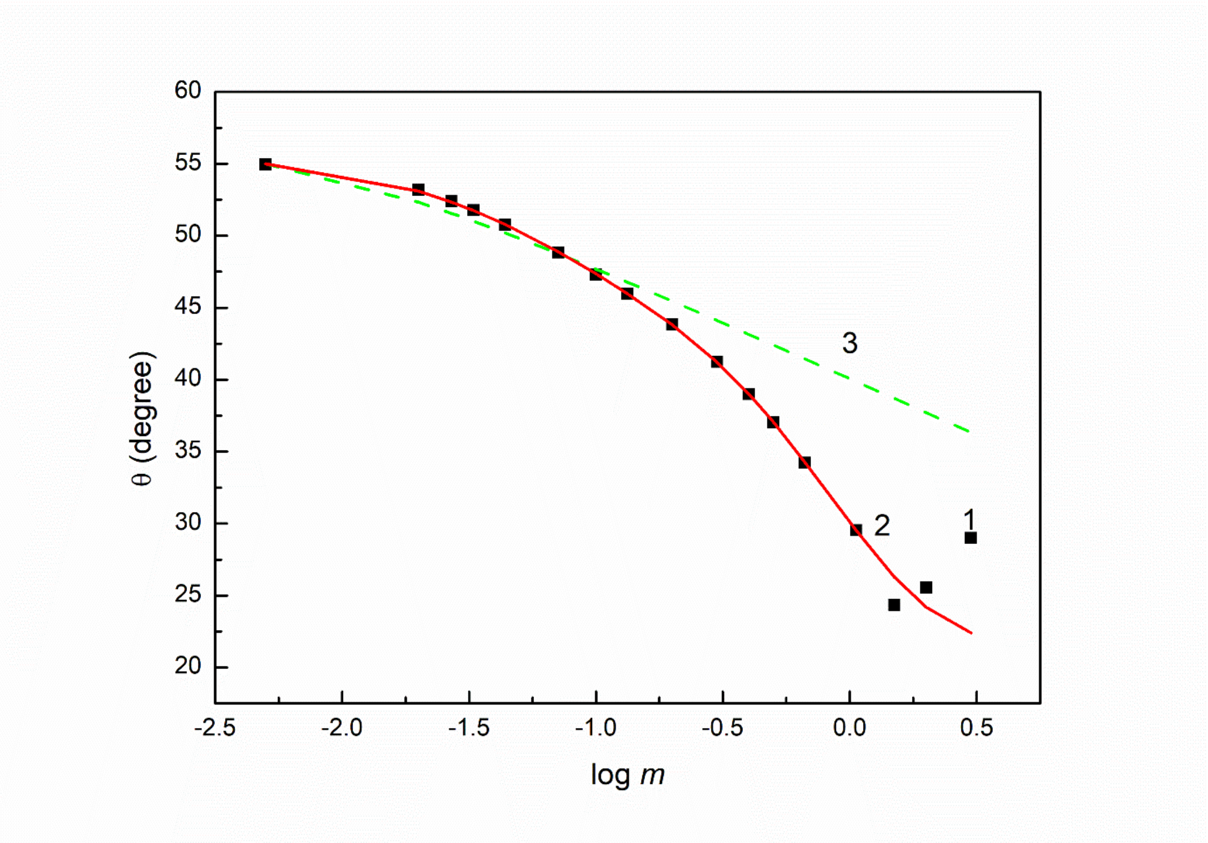

Figure S20. A plot of the contact angle (θ) measured for the aqueous solution of fraction E0 on the glass surface (points 1) and calculated from Equation (6) (curve 2) and Equation (7) (curve 3) vs. the logarithm from the fraction concentration (log m).

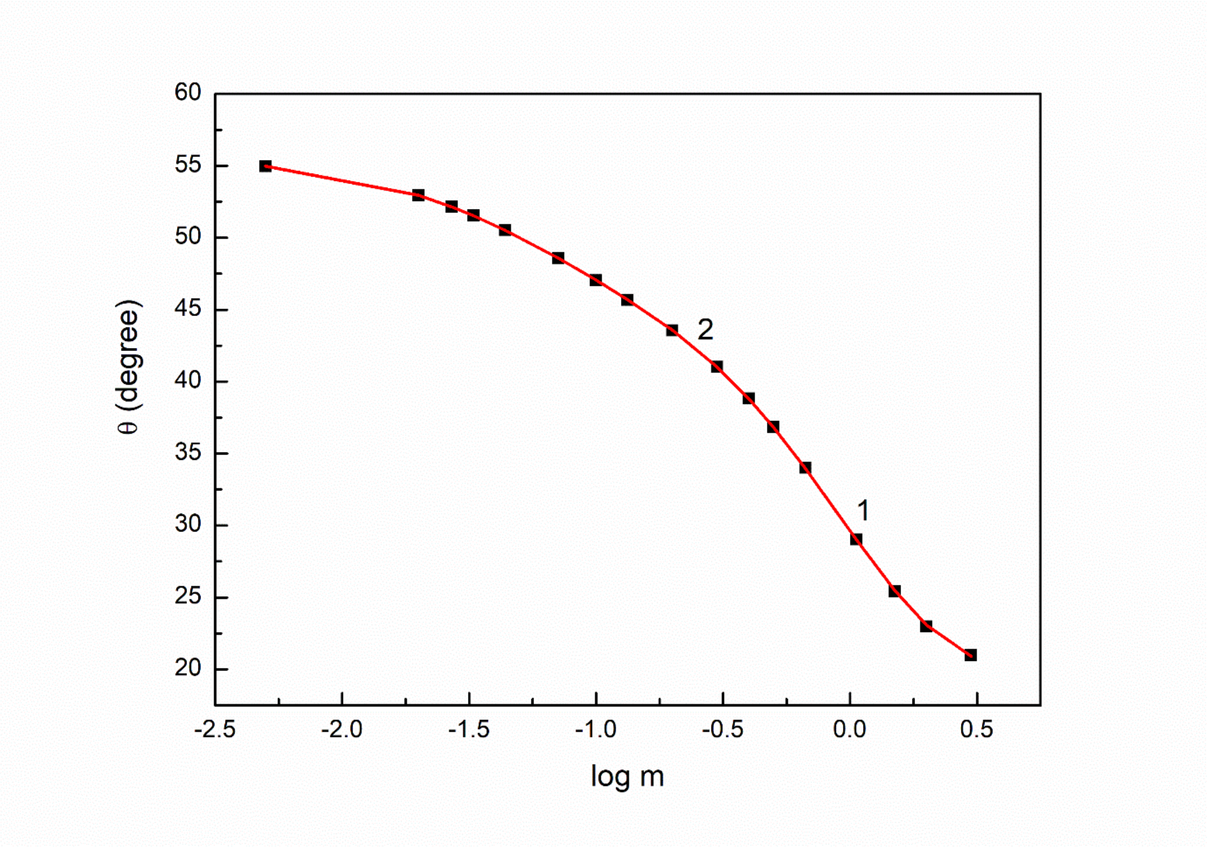

Figure S21. A plot of the contact angle (θ) measured for the aqueous solution of fraction E1 on the glass surface (points 1) and calculated from Equation (6) (curve 2) vs. the logarithm from the fraction concentration (log m).

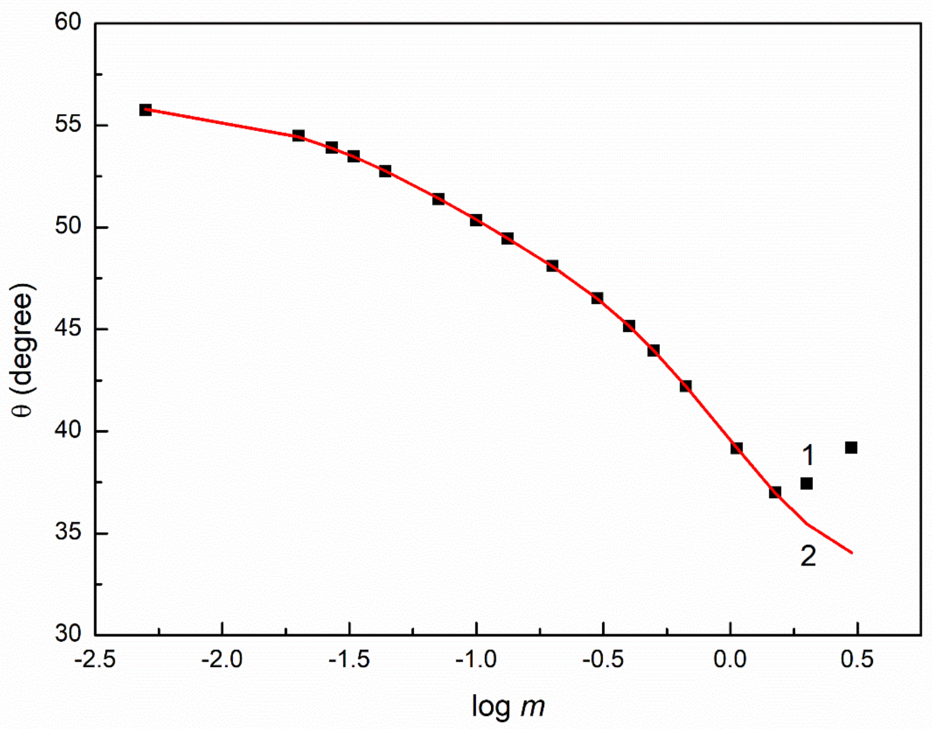

Figure S22. A plot of the contact angle (θ) measured for the aqueous solution of fraction E2 on the glass surface (points 1) and calculated from Equation (6) (curve 2) vs. the logarithm from the fraction concentration (log m).

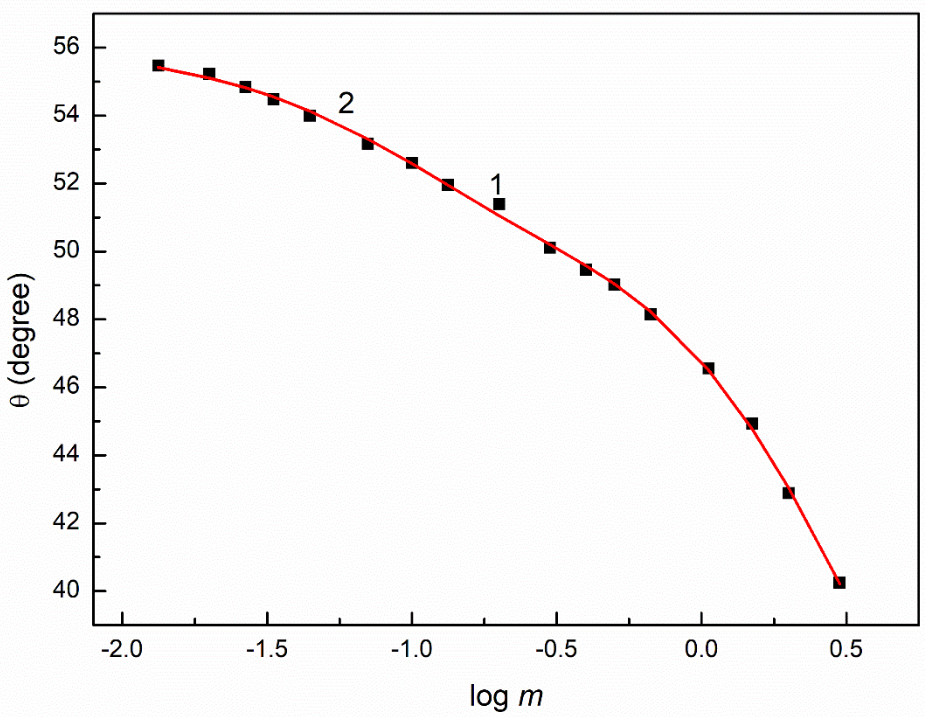

Figure S23. A plot of the contact angle (θ) measured for the aqueous solution of fraction E3 on the glass surface (points 1) and calculated from Equation (6) (curve 2) vs. the logarithm from the fraction concentration (log m).

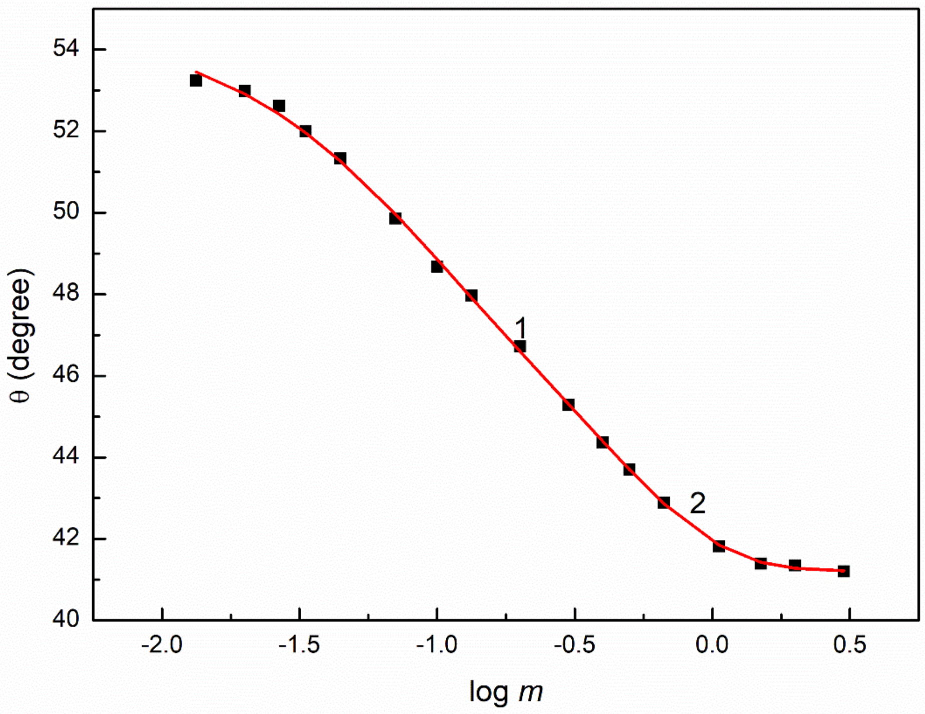

Figure S24. A plot of the contact angle (θ) measured for the aqueous solution of fraction E4 on the glass surface (points 1) and calculated from Equation (6) (curve 2) vs. the logarithm from the fraction concentration ($\log m$).

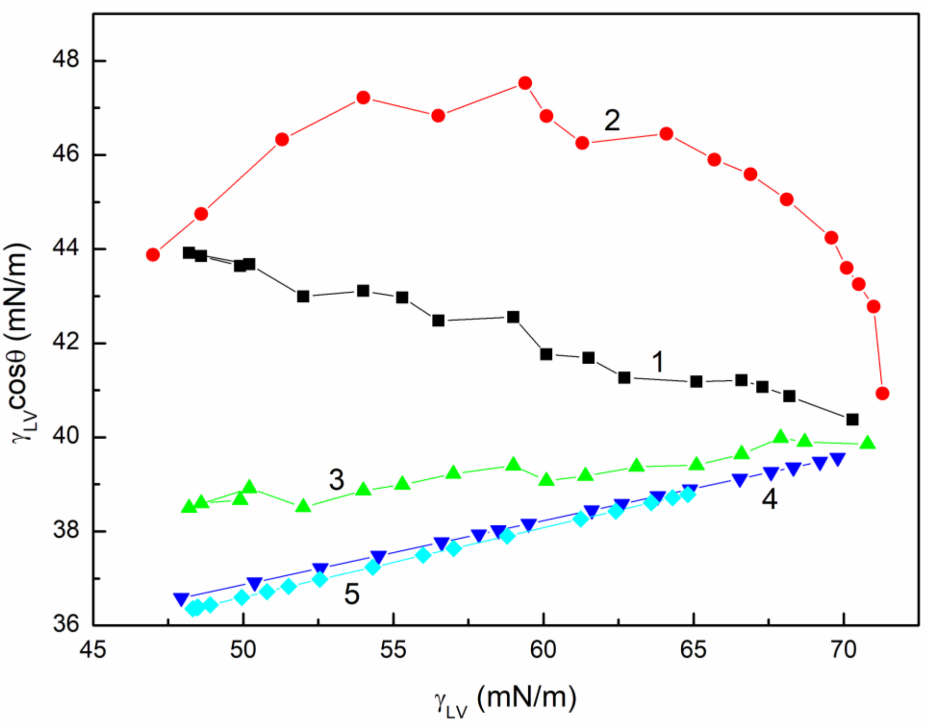

Figure S25. A plot of the adhesion tension ($\gamma_{LV}\cos\theta$) vs. the aqueous solution surface tension (γ_{LV}) for glass. Curves 1 – 5 correspond to the fraction E0, E1, E2, E3 and E4, respectively.

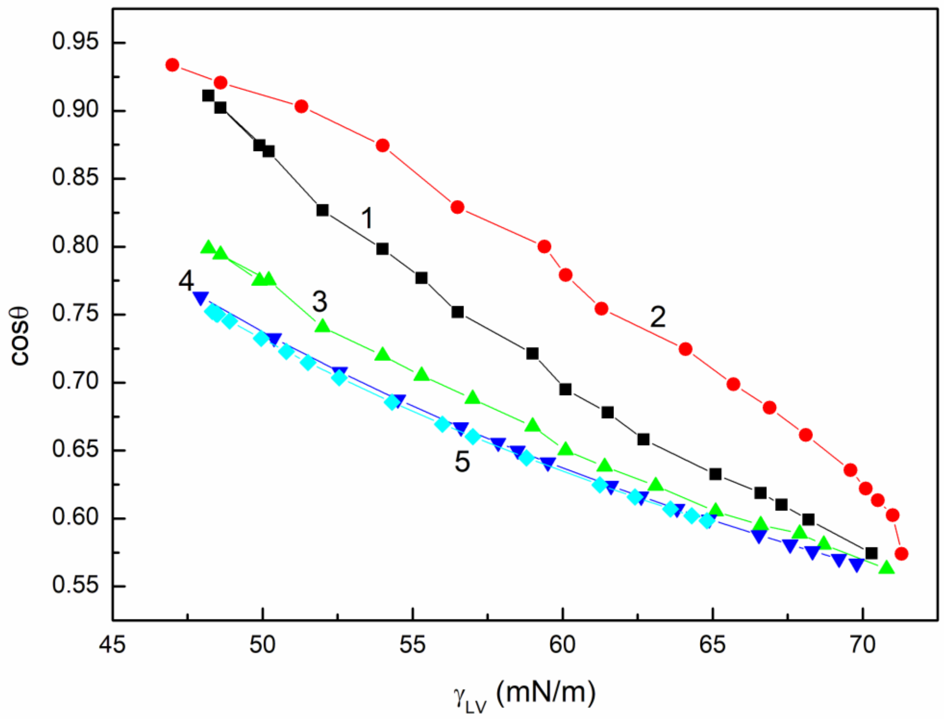

Figure S26. A plot of the cosine of the contact angle vs. the aqueous solution surface tension (γ_{LV}) for PMMA. Curves 1 – 5 correspond to the fraction E0, E1, E2, E3 and E4, respectively.

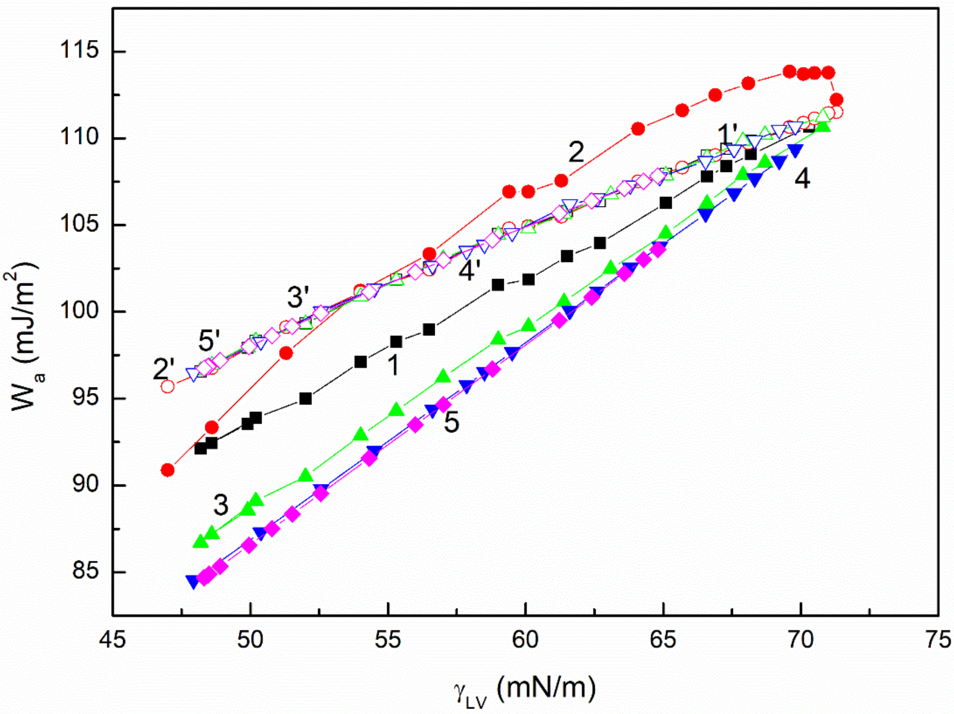

Figure S27. A plot of the adhesion work (W_a) of the solution to the glass surface calculated from the expression $W_a = 2\sqrt{\gamma_{LV}^{LW}\gamma_{SV}^{LW}} + 2\sqrt{\gamma_{LV}^{+}\gamma_{SV}^{-}}$ (curves 1 – 5) and from $W_a = \gamma_{LV}(\cos\theta + 1)$ (curves 1' – 5') vs. the solution surface tension (γ_{LV}). Curves 1 – 5 as well as 1' – 5' correspond to the fraction E0, E1, E2, E3 and E4, respectively.

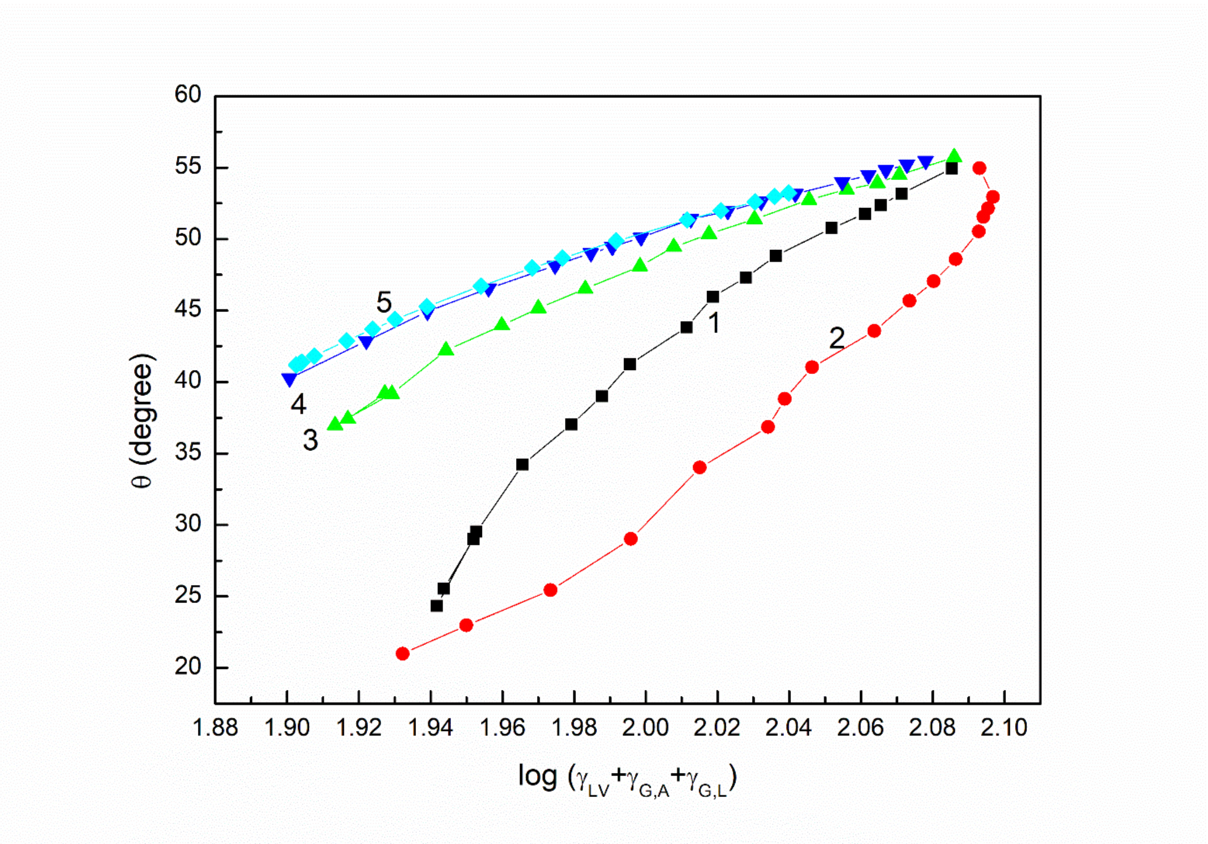

Figure S28. A plot of the contact angle (θ) measured on the glass surface vs. the logarithm of the sum of the solution and PMMA surface tension and solid-liquid interface tension ($\gamma_{LV} + \gamma_{G,A} + \gamma_{G,L}$). Curves 1 – 5 correspond to the fraction E0, E1, E2, E3 and E4, respectively

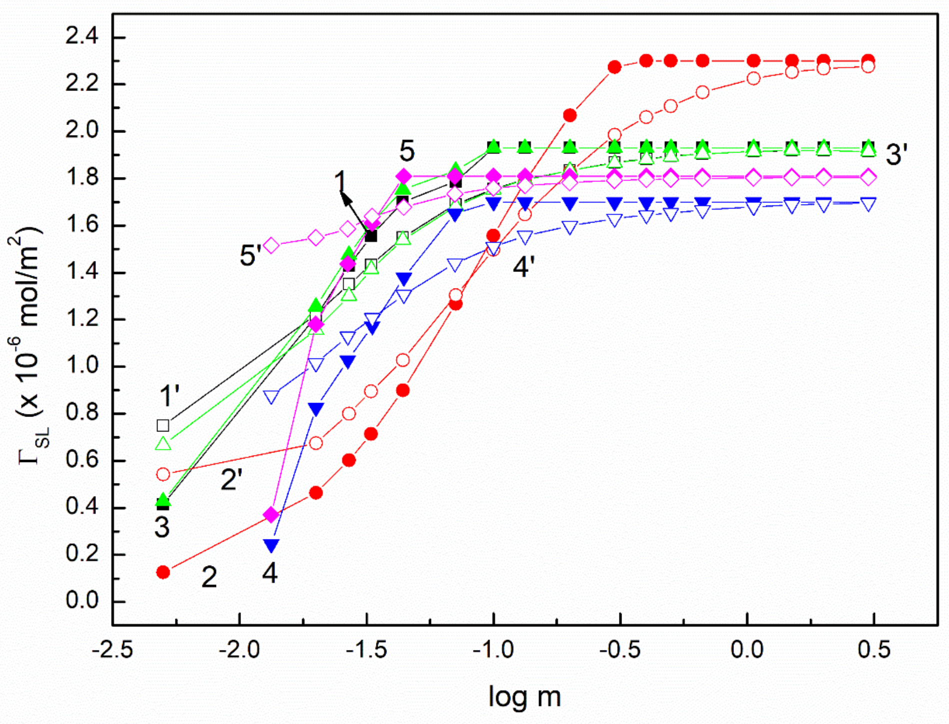

Figure S29. A plot of the surface concentration at the PTFE-solution interface (Γ_{SL}) calculated from the Gibbs (curves 1 – 5) and modified Langmuir equations (11) (curves 1' – 5') vs. the logarithm from the fraction concentration ($\log m$). Curves 1 – 5 as well as 1' – 5'correspond to the fraction E0, E1, E2, E3 and E4, respectively.

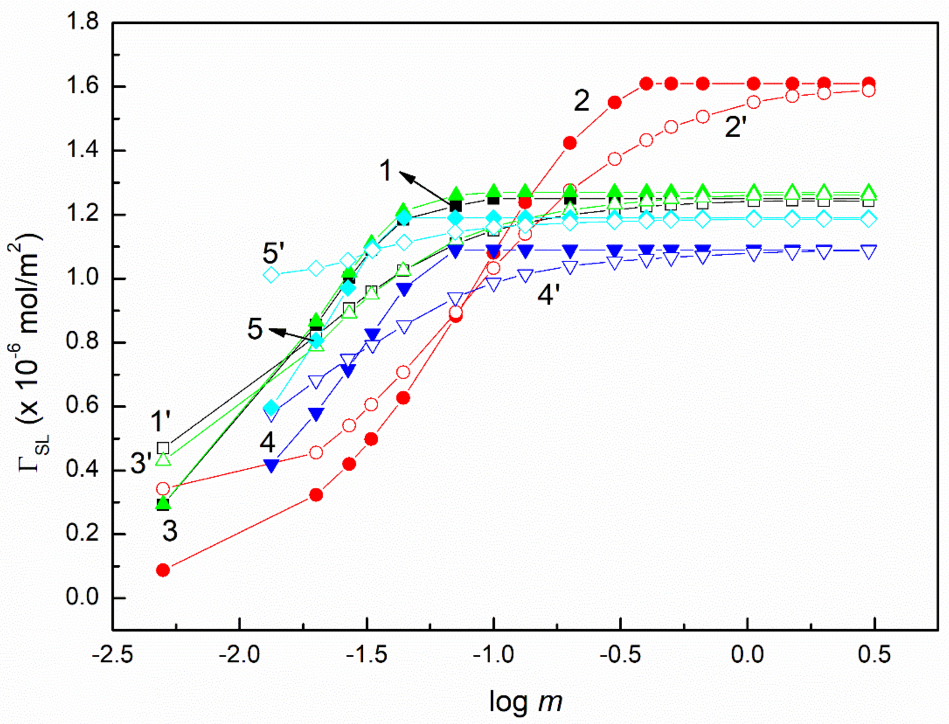

Figure S30. A plot of the surface concentration at the PMMA-solution interface (Γ_{SL}) calculated from the Gibbs (curves 1 – 5) and modified Langmuir equations (curves 1' – 5') vs. the logarithm from the fraction concentration ($\log m$). Curves 1 – 5 as well as 1' – 5' correspond to the fraction E0, E1, E2, E3 and E4, respectively.

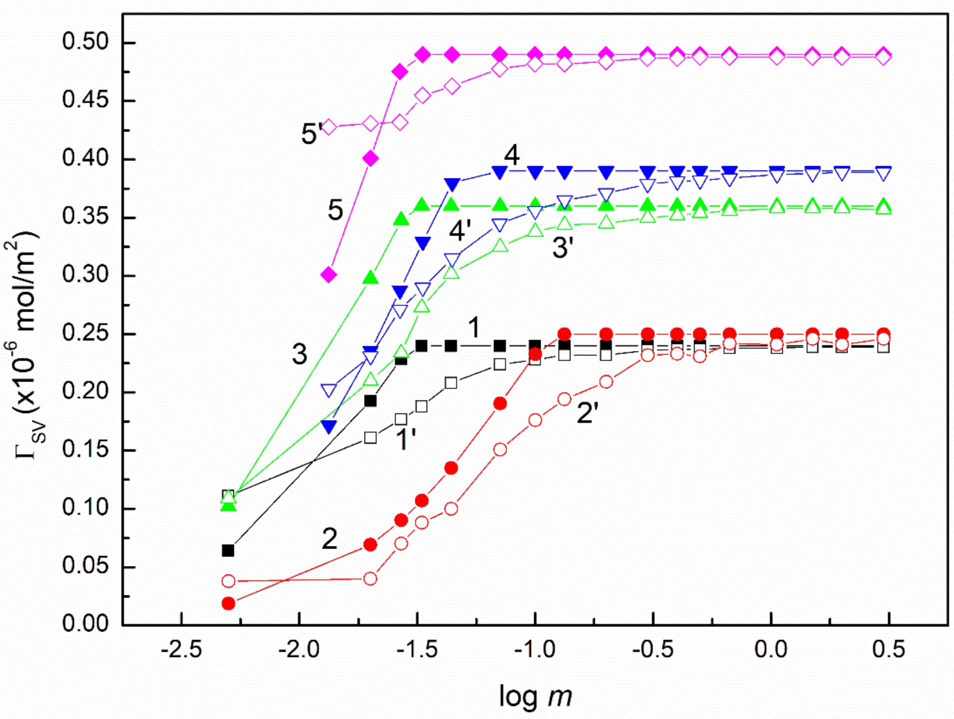

Figure S31. A plot of the surface concentration at the PMMA-air interface (Γ_{SV}) calculated from the Gibbs (13) (curves 1 – 5) and modified Langmuir equations (curves 1' – 5') vs. the logarithm from the fraction concentration ($\log m$). Curves 1 – 5 as well as 1' – 5' correspond to the fraction E0, E1, E2, E3 and E4, respectively.

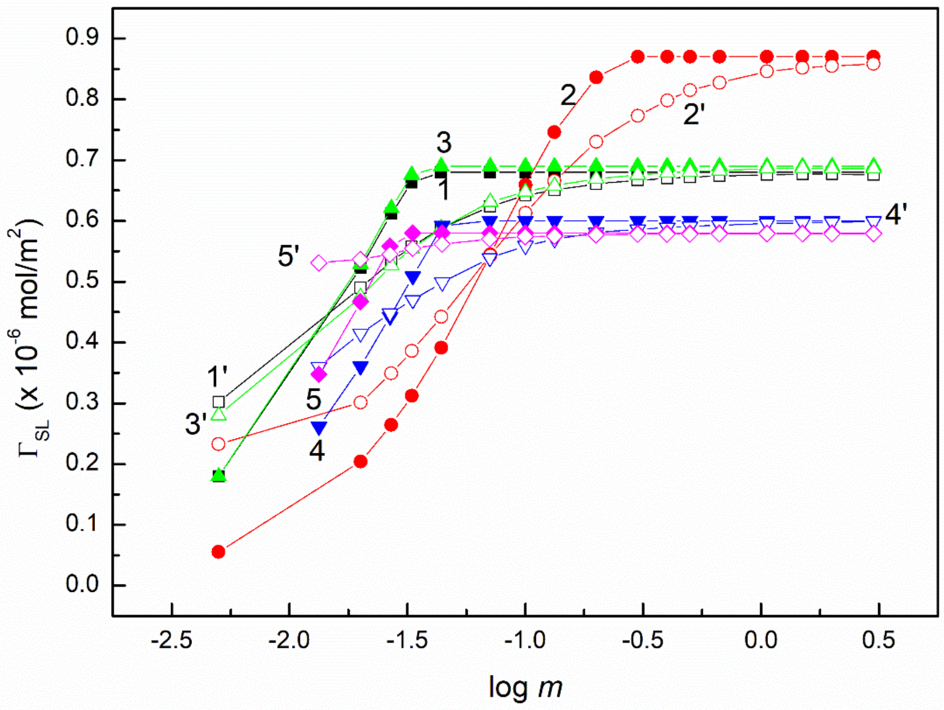

Figure S32. A plot of the surface concentration at the glass-solution interface (Γ_{SL}) calculated from the Gibbs (curves 1 – 5) and modified Langmuir equations (curves 1' – 5') vs. the logarithm from the fraction concentration ($\log m$). Curves 1 – 5 as well as 1' – 5' correspond to the fraction E0, E1, E2, E3 and E4, respectively.

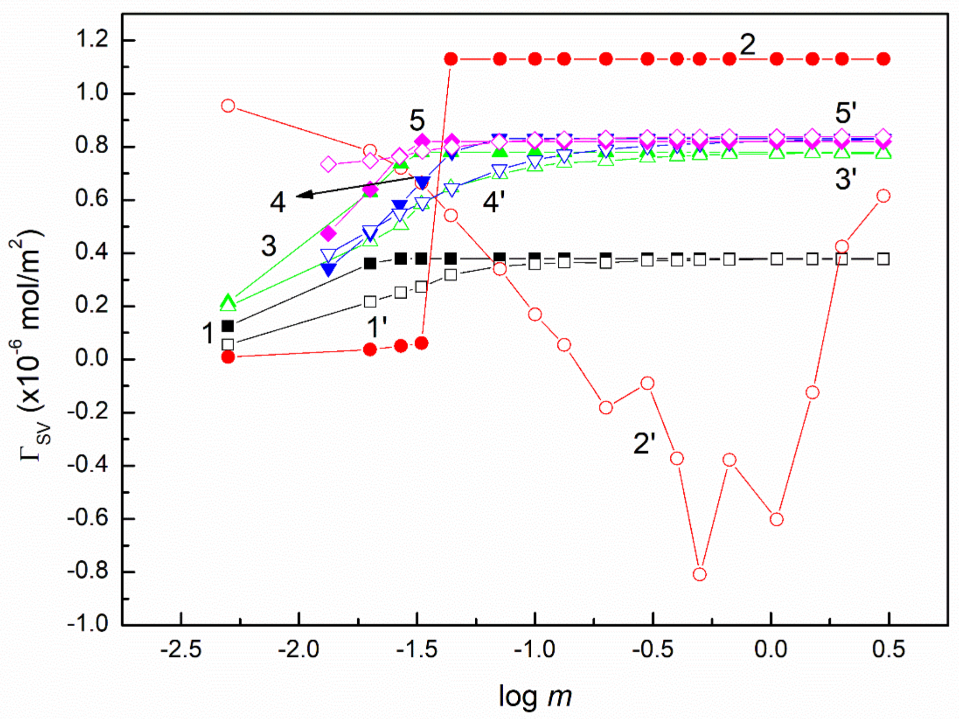

Figure S33. A plot of the surface concentration at the glass-air interface (Γ_{SV}) calculated from the Gibbs (curves 1 – 5) and modified Langmuir equations (curves 1' – 5') vs. the logarithm from the fraction concentration ($\log m$). Curves 1 – 5 as well as 1' – 5' correspond to the fraction E0, E1, E2, E3 and E4, respectively.
